# TOR-inhibitor insensitive-1 (TRIN1) regulates cotyledons greening in *Arabidopsis*

**DOI:** 10.3389/fpls.2015.00861

**Published:** 2015-10-19

**Authors:** Linxuan Li, Yun Song, Kai Wang, Pan Dong, Xueyan Zhang, Fuguang Li, Zhengguo Li, Maozhi Ren

**Affiliations:** ^1^School of Life Sciences, Chongqing UniversityChongqing, China; ^2^Institute of Cotton Research, Chinese Academy of Agricultural Sciences, the State Key Laboratory of Cotton BiologyHenan, China

**Keywords:** *TRIN1*, TOR, TOR kinase inhibitors, cotyledons greening, *Arabidopsis*

## Abstract

Target of Rapamycin (TOR) is an eukaryotic protein kinase and evolutionally conserved from the last eukaryotic common ancestor (LECA) to humans. The growing evidences have shown that TOR signaling acts as a central controller of cell growth and development. The downstream effectors of TOR have been well-identified in yeast and animals by using the immunosuppression agent rapamycin. However, less is known about TOR in plants. This is largely due to the fact that plants are insensitive to rapamycin. In this study, AZD8055 (AZD), the novel ATP-competitive inhibitor of TOR, was employed to decipher the downstream effectors of TOR in *Arabidopsis*. One AZD insensitive mutant, ***T****O****R****-****i****nhibitor i****n****sensitive-****1*** (*trin1*), was screened from 10,000 EMS-induced mutation seeds. The cotyledons of *trin1* can turn green when its seeds were germinated on ½ MS medium supplemented with 2 μM AZD, whereas the cotyledons greening of wild-type (WT) can be completely blocked at this concentration. Through genetic mapping, *TRIN1* was mapped onto the long arm of chromosome 2, between markers SGCSNP26 and MI277. Positional cloning revealed that *TRIN1* was an allele of *ABI4*, which encoded an ABA-regulated AP2 domain transcription factor. Plants containing *P35S::TRIN1* or *P35S::TRIN1-GUS* were hypersensitive to AZD treatment and displayed the opposite phenotype observed in *trin1*. Importantly, GUS signaling was significantly enhanced in *P35S::TRIN1-GUS* transgenic plants in response to AZD treatment, indicating that suppression of TOR resulted in the accumulation of TRIN1. These observations revealed that TOR controlled seed-to-seedling transition by negatively regulating the stability of TRIN1 in *Arabidopsis*. For the first time, TRIN1, the downstream effector of TOR signaling, was identified through a chemical genetics approach.

## Introduction

TOR, which belongs to the phosphoinositide 3-kinase (PI3K)-related kinase family, is an evolutionarily conserved Ser/Thr kinase in structure and function from the last eukaryotic common ancestor (LECA) to humans. TOR regulates cell growth, metabolism and development in response to dynamic and diverse environmental stresses and challenges (Horváth et al., 2006; Wullschleger et al., 2006; Laplante and Sabatini, 2012; Ren et al., 2012; Robaglia et al., 2012; Yuan et al., 2013). Two *TOR* genes were originally identified by genetic screening for rapamycin insensitive mutants in budding yeast (*Saccharomyces cerevisiae*) (Heitman et al., 1991b; Cafferkey et al., 1993; Kunz et al., 1993). Subsequently, only one *TOR* gene was identified in animals, humans and *Arabidopsis* (Sabatini et al., 1994; Sabers et al., 1995; Menand et al., 2002). TOR protein contains five conserved regions: HEAT repeats, FAT domain, FRB domain, kinase domain and FATC domain (Menand et al., 2002; Mahfouz et al., 2006). In yeast and mammals, TOR forms two structurally and functionally distinct protein complexes: TORC1 (TOR complex 1) and TORC2 (TOR complex 2; Loewith et al., 2002). TOR, regulatory-associated protein of mTOR (RAPTOR) and lethal with SEC13 protein 8 (LST8) constitute the core of TORC1, which regulates cell growth and metabolism in response to nutrients and energy requirements (Martin and Hall, 2005; Wang and Proud, 2009). Rapamycin can specifically bind to FK506 binding protein 12 (FKBP12), which interacts with the FRB domain of TOR and forms a rapamycin-FKBP12-TOR complex to inhibit the activity of TORC1 in yeast and animals (Heitman et al., 1991b; Zheng et al., 1995). On the other hand, TORC2 is insensitive to rapamycin and the core components include TOR, LST8 and rapamycin-insensitive companion of mTOR (RICTOR). TORC2 controls spatial cell growth by regulating cytoskeletal structure and polarity (Sarbassov et al., 2004; De Virgilio and Loewith, 2006; Wullschleger et al., 2006). It seems that the components of TORC2 are much less conserved across the eukaryotic species than that of TORC1, which suggests that the functions of TORC2 may likely vary across species. For example, RAPTOR, the core member of TORC1, has been identified in *Arabidopsis*, but the homologs of RICTOR, the key defining effector of TORC2, are missing in most examined plants (Xiong and Sheen, 2014; Rexin et al., 2015), suggesting that plants most likely have the distinct TORC2 throughout evolution.

A recent study showed that TOR could directly phosphorylate transcription factor E2Fa to activate S-phase genes in root meristems (Xiong and Sheen, 2013), and this finding revealed an important role of TOR in the regulation of cell cycle. TOR also played an essential role in the regulation of primary and secondary metabolism in plants. Disruption of TOR by reduction of *TOR* expression or kinase activity led to the accumulation of high levels of starch, triacylglycerides, amino acids, TCA intermediates and secondary metabolites (Deprost et al., 2007; Ren et al., 2012; Caldana et al., 2013). Genetic and physiological studies combining with large-scale transcript and metabolite profiling analyses have revealed that TOR regulates plant growth, development, flowering, senescence and life span by modulating transcription, translation, cell cycle, autophagy and metabolism (Deprost et al., 2007; Ahn et al., 2011; Ren et al., 2011, 2012; Moreau et al., 2012; Xiong and Sheen, 2012, 2014; Caldana et al., 2013).

The lesions of TOR result in lethality in yeast, animals and plants (Heitman et al., 1991a; Menand et al., 2002; Ren et al., 2011). This severely prevented people from identifying the downstream effectors of TOR signaling through classic genetic approaches. Significant discoveries on the functions of TORC1 did not occur until rapamycin was found and applied for the study of TOR in yeast and animals (Heitman et al., 1991a; Brown et al., 1994). Rapamycin is the first generation of TOR inhibitors, and it inhibits the activity of TORC1 only in the presence of FKBP12. Although FKBP12 is a non-essential protein for cell growth, it plays a key role in mediating the cytotoxicity of rapamycin on cell growth. Large amount of information about TORC1 and its downstream targets have been well documented in yeast and animals (Burnett et al., 1998; Nojima et al., 2003; Martin et al., 2004; Ahn et al., 2011). However, information on TOR signaling in plants is limited, which is mainly due to its insensitivity to rapamycin (Xu et al., 1998). Plants are anchored in soil and rapamycin is produced by the soil bacterium *Streptomyces hygroscopicus*. To escape from rapamycin inhibition, plants have adapted an evolutionary mutation in the *FKBP12* gene that results in its loss of function to bind rapamycin and thus fail to mediate rapamycin to inhibit TOR activity (Xu et al., 1998; Sormani et al., 2007). To assess TOR signaling in plants by using rapamycin, Sormani et al. and Ren et al. independently generated rapamycin-hypersensitive plants by introducing yeast or human *FKBP12* into *Arabidopsis* (Sormani et al., 2007; Ren et al., 2012). However, rapamycin inhibits the activity of TOR only in the presence of yeast or animals *FKBP12* in plants (Sormani et al., 2007; Ren et al., 2012). This largely restricts the usage of rapamycin on various plants. In addition, previous studies have shown that rapamycin cannot fully inhibit the growth of plants harboring exogenous *FKBP12* even at a concentration of 20 μM (Ren et al., 2012), which is 2000-fold greater than the dosage used in yeast and animals, suggesting that rapamycin partially inhibits TOR signaling and the broader functions of TOR cannot be deciphered by using rapamycin alone in plants.

To resolve this issue relating to rapamycin, active-site TOR inhibitors (asTORis), which are the second generation inhibitors of TOR, are well developed in mammalian cells. Unlike rapamycin, asTORis which include PP242, Torin1, Torin2, AZD, and KU63794 can directly and specifically target the ATP-binding pocket of the TOR kinase domain to suppress both the functions of TORC1 and TORC2 by competing with ATP in mammalian cells (Feldman et al., 2009; García-Martinez et al., 2009; Chresta et al., 2010; Liu et al., 2011). Recent studies have shown that AZD displayed highly selective and inhibitory effects on TOR activity in flowering plants, including *A. thaliana, O. sativa*, and *L. japonicas* (Calabrese and Blain, 2009; Montané and Menand, 2013). Importantly, the lesser copy numbers of TOR, the more inhibitory effects of AZD was observed in *Arabidopsis* (Calabrese and Blain, 2009; Montané and Menand, 2013). Based on these observations, in this study, we generated an EMS-induced mutation library and identified a *trin1* mutant from this library through large-scale genetic screening and positional cloning. We found that TRIN1 acted as a negative effector of TOR signaling to modulate cotyledons greening in *Arabidopsis*. For the first time, the downstream target of TOR signaling was identified by using asTORis-based chemical genetics approaches.

## Materials and methods

### Plant materials and growth conditions

In this study, WT *Arabidopsis* Columbia (Columbia-0), Landsberg *erecta* (L*er*) ecotype and *abi4-1* were used. *Arabidopsis* seeds were surface sterilized by using liquid methods. The seeds first treated with 70% ethanol for 2 min and the supernatant was discarded; then, with 10% sodium hypochlorite containing 0.3% Tween-20 for 5 min, and the supernatant was discarded; followed by four or five rinses with sterile water, centrifugation for 2 min at 4000 g each time, and the supernatant was discarded. Finally, the seeds were suspended in 0.1% sterile agarose and kept at 4°C for 2 days. Sterilized seeds were plated on plates, and then grown in a controlled environment at 22°C under 16 h 60–80 μE·m^−2^ s^−1^ continuous light and 8 h darkness.

### EMS mutagenesis of *Arabidopsis* col WT seeds and genetic mapping

A total of ~10,000 seeds were placed in a 50-mL centrifuge tube and ultrapure water was added to about 1 cm above the seeds. The seeds were soaked at room temperature for 12 h. Water was decanted and 20 mL 0.3% ethyl methanesulfonate (EMS) (v/v) in water was added. The seeds were incubated for 15 h at room temperature (shock was introduced every hour), followed by decanting of the EMS and rinsing with 40 mL ultrapure water (5 times, 5 min each). The seeds were sown in soil and grown in the greenhouse until maturity.

For mapping, *trin1* homozygous mutants were crossed with WT Landsberg *erecta* (L*er*), and F_2_ seeds were obtained. Genomic DNA was isolated from the F_2_ plants that exhibited the AZD-insensitive phenotype, and the gene was mapped using simple sequence repeat (SSR) markers.

### Generation of overexpression constructs and transformation of *Arabidopsis* plants

Total DNA was extracted from *Arabidopsis* Col WT seedlings using the DNAprep Pure Plant Kit (BioTeke, Beijing, China). The promoter of *TRIN1* (2051 bp in length), full-length CDS of *TRIN1* (987 bp), and *GUS* (1812 bp) were amplified by RT-PCR using the TransStar Taq Polymerase Mix kit (TRANSGEN, Beijing, China) following the manufacturer's instructions. The corresponding restriction enzyme sites were introduced into 5′ and 3′ end of the respective primer (Supplemental Table 1). The PCR products of the promoter of *TRIN1* (*PTRIN1*) and CDS of *TRIN1* and *GUS* were cloned into the TA cloning vector pEASY-T1 Simple (TRANSGEN, Beijing, China) and verified by DNA sequencing. Then the *GUS* coding sequence was subcloned downstream of the *TRIN1* promoter or coding sequence (Supplemental Figure 1), and the *TRIN1* coding sequence was subcloned downstream of the cauliflower mosaic virus *35S* promoter in vector p8GWN to generate clones *P35S::TRIN1-HA, P35S::TRIN1-GUS, P35S::GUS* and *PTRIN1::GUS* (Supplemental Figure 1). A gateway system–based entry vector was generated by cloning the recombinant plasmids into p8GWN (Ren et al., 2011). These recombinant constructs were transformed into pEarleyGate303 (Earley et al., 2006) through LR recombination reactions (Supplemental Figure 1).

The constructs were introduced into *Agrobacterium tumefaciens* strain LBA4404 and used in the transformation of WT Col plants. Transgenic *Arabidopsis* lines were generated by *Agrobacterium* mediated transformation using the floral dip method (Zhang et al., 2006) for developmental and phenotypic analyses. Transgenic lines were selected on ½ MS medium containing 50 mg/L kanamycin. Plants were allowed to reproduce for two generations, and the T3 homozygous plants were used in the analysis.

### Quantitative real-time PCR

Total RNA of *trin1, TRIN1*-OE5, and WT seedlings that were treated for 36 h in mediums containing DMSO, AZD (5 μM), and Torin1 (10 μM) was isolated using the RNAprep Pure Plant Kit (TIANGEN, Beijing, China). Total RNA was treated with RNase-free DNase (Promega). PrimeScript® RT reagent kit (Takara, Dalian, China) was used for reverse transcription, following the manufacturer's instructions. Relative transcript levels were assayed by one-step real-time PCR analysis using the CFX96 real-time PCR system (BIO-RAD, USA). Real-time primers were designed by Primer Premier 5.0 and the details are presented in Supplemental Table 2. *AtACTIN2* was used as an internal control. Reactions were performed in a final volume of 20 μL containing 10 μL of 2 × Power Top Green qPCR SuperMix (TRANSGEN, Beijing, China), 50 ng of cDNA, and 500 nM of each of the forward and reverse primers. The following default program was used: 94°C for 5 min, followed by 40 cycles of 94°C for 15 s and 60°C for 30 s each, and a dissociation stage of 95°C for 15 s, 60°C for 30 s, and 95 °C for 15 s. RNA relative quantification analyses were performed with the Bio-Rad CFX Manager software. The data were expressed as the mean ± SD of three independent experiments. Each data point was determined in triplicate in each of the three biological replicates and expressed as the mean ± SD.

### GUS staining and quantitative determination of GUS activity

Transgenic plants staining for GUS activity using X-Gluc were performed as previously described (Menand et al., 2002). The first true leaves of the *P35S::TRIN1-GUS* OE-2 plants were incubated with GUS staining solution [50 mM NaOH, 0.5 mM 5-bromo-4-chloro-3-indolyl-β-D-glucuronide, 0.5 mM K3Fe(CN)6, 0.5 mM K4Fe(CN)6, and 0.05% (v/v) Triton X-100 (pH 7.5)] for 8–12 h at 37°C, and a 70% ethanol wash was performed to remove chlorophyll from the leaves. Images were captured using a stereomicroscope (OLYMPUS MVX10, Japan). Each treatment was performed using three biological replicates.

Quantitative GUS assay was performed using the MarkerGene™ β-glucuronidase (GUS) reporter gene activity detection kit (Marker Gene Technologies, Inc., Eugene, OR, USA). Total proteins in extracts of the first true leaves were quantified using the Bradford assay (Bradford, 1976). Fluorometric quantification of GUS activity was performed using 4-methylumbelliferone (4-MU) substrate, and fluorescence was determined on a Tecan 200 fluorometer (Tecan, Durham, NC, USA) using 360 and 465 nm as excitation and emission wavelengths, respectively. GUS activity of the extracts was calculated in nmol 4-MU per minute per mg protein.

### Measurement of chlorophyll content and shoots

Chlorophyll content was measured after treatments using the specified agents for 0–6 days. Chlorophyll was extracted from the plant leaves and quantified (Weaver and Amasino, 2001). *Arabidopsis* seeds were plated on ½ MS culture medium containing the specified agents, and the length of emerging shoots were measured after 10 days. Three biological experiments, each consisting of 30 plants per treatment were measured.

## Results

### AZD is able to block seed-to-seedling transition in *Arabidopsis*

**Figure 1:**
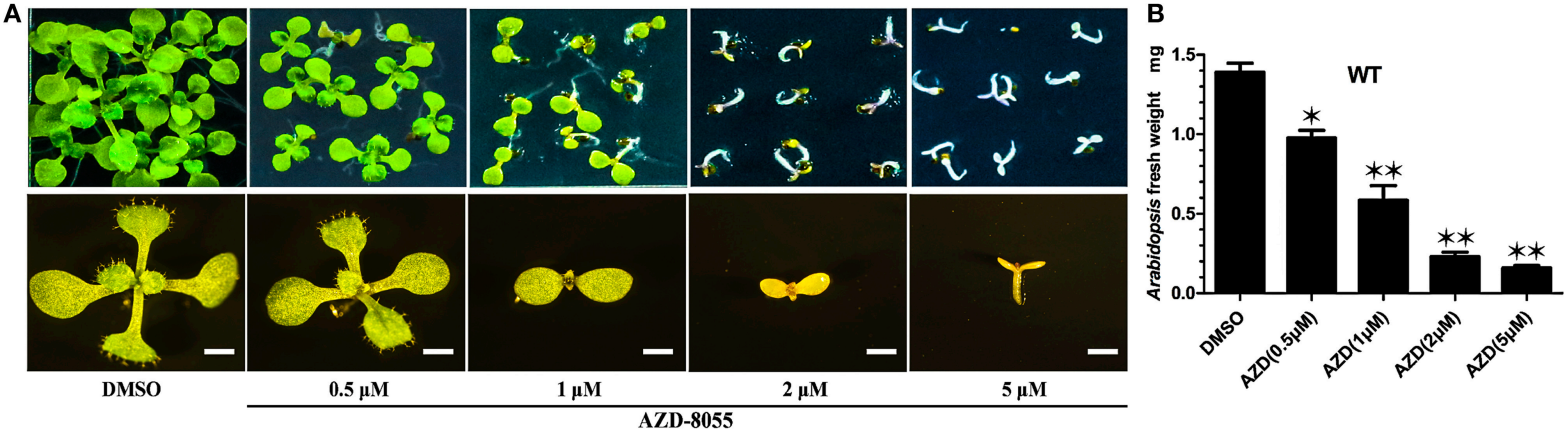
**AZD arrests early seedlings establishment in *Arabidopsis***. **(A)** Representative images of WT plants growing on ½ MS medium containing increasing concentrations of AZD for 10 days (upper panel). The lower panel shows enlarged images of the individual seedling in the upper panel. Bar = 1 mm. **(B)** Fresh weight of WT seedlings growing on different AZD concentrations for 10 days. Each graph represents the average of 30 seedlings that were conducted in triplicate. Error bars indicate ±SD for triplicates. Asterisks denote Student's *t*-test significant difference compared with DMSO (^*^P < 0.05; ^**^P < 0.01).

TOR protein is a highly conserved Ser/Thr kinase protein. Among all the (*Arabidopsis thaliana* TOR) *At*TOR functional domains, including HEAT repeats, FAT, FRB, kinase, and FATC domains, the kinase domain of *At*TOR showed the highest amino acid sequence identical to that of yeast and human (Supplemental Figure 2). AZD, a novel ATP competitive inhibitor of mTORC1 and mTORC2, can directly bind to the kinase domain of TOR to compete with ATP (Chresta et al., 2010). Montane and Menand showed that AZD could specifically inhibit the TOR activity in *Arabidopsis* (Supplemental Figure 3A), resulting in short roots and shoots, bleached cotyledons and a severe delay in seedling development (Montané and Menand, 2013). To identify the downstream components of the TOR signaling pathway in *Arabidopsis*, AZD was applied to screen AZD-insensitive mutant seeds derived from the EMS mutagenesis library in this study. To determine the optimal AZD concentration for mutants screening, WT seeds were treated with different concentrations of AZD dissolved in DMSO. Consisted with the findings of previous reports, AZD inhibited seedling growth and development in a dose-dependent manner. With increasing concentrations of AZD, the developing seedlings were subjected to different degrees of inhibition (Figure 1A). When applied concentration of AZD reached to 1 μM, the fresh weight of seedlings was only half of that treated with DMSO control (Figure 1A), suggesting that 1 μM AZD can be used as the 50% growth inhibitory dose (GI_50_) of AZD in *Arabidopsis* (Figure 1B). Additionally, the cotyledons did not turn green and the seedlings completely ceased to grow and develop with 2 μM AZD. Thus, 2 μM AZD was selected as the optimal concentration for screening AZD-insensitive mutant seeds.

### Screening, genetic analysis and fine mapping of trin1 mutant

Approximately 100,000 EMS-induced M_2_ mature seeds were harvested and then cultured on ½ MS medium (Murashige and Skoog, 1962) containing 2 μM AZD. Because seed-to-seedling transition of WT plants can be blocked by using 2 μM AZD, the 15 days after germination greening seedlings grown on 2 μM AZD medium were selected as AZD-insensitive mutants for further study. Nine independent AZD-insensitive mutants in total were obtained from this screening and the first of these mutants was named as ***T****O****R****-****i****nhibitor i****n****sensitive-****1*** (*trin1*).

The *trin1* mutant was backcrossed with Col-0 WT plants for three times. The results consistently showed that the AZD-insensitive phenotype was absent in every F_1_ generation of hybrid plants, and the F_2_ population segregated into WT and mutant at a ratio of 3:1 (Table 1), indicating that the AZD-insensitive phenotype was caused by a monogenic recessive mutation. The *trin1* homozygous mutant plants were crossed with *Arabidopsis* L*er* plants to generate the segregation population for fine mapping of the genetic loci of *trin1*.

**Table 1:** **Genetic segregation and complementation tests**.

**Cross**	**Population**	***N*_total_**	***N*_mutant_**	***N*_wt_**	**Ratio**
*trin1* × Col-0	F_1_	24	0	24	0
*trin1* × Col-0	F_2_	316	83	233	1:3
*TRIN1* × *trin1*	F_1_	41	0	41	0
*TRIN1* × *trin1*	F_2_	1296	328	968	1:3
*trin1* × *abi4-1*	F_1_^*^	17	17	0	8

Seeds (N_total_) were cultured on ½ MS medium containing 2 μM AZD, and the number of trin1 mutant (N_mutant_) and WT (N_wt_) was scored after 7 days. F_1_

* *refers to the seeds of the F_1_ population which were generated by crossing trin1 mutant with abi4-1 were cultured on ½ MS medium containing 1 μM ABA instead of AZD*.

**Figure 2:**
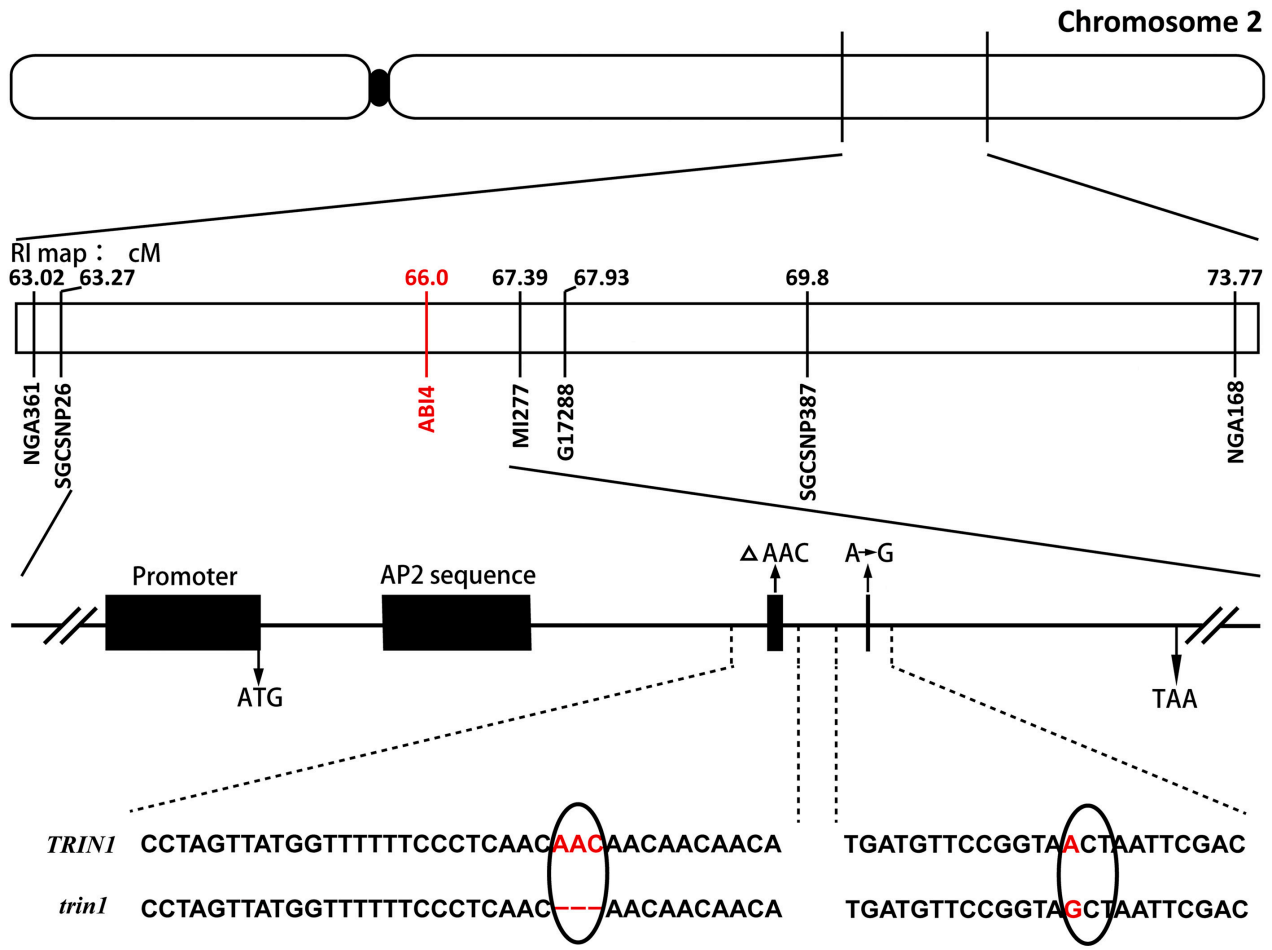
**Fine mapping of *TRIN1* on chromosome 2**. Sequence analysis of *trin1* shows a 3-bp (AAC) deletion (as indicated by a Δ) at positions 566–568 in the coding sequence that leads to deletion of the amino acid asparagine. Furthermore, a point mutation changing the first base of codon 229 of *trin1* from A to G was identified (marked in red); it results in the substitution of threonine (ACT) with an alanine (GCT). The black solid oval represents the centromere.

Genomic DNA was isolated from 500 F_2_ plants that exhibited the AZD-insensitive phenotype, and the mutation was roughly mapped to chromosome 2 between SSR markers nga361 and nga168. More markers between nga361 and nga168 were designed. A candidate region with the mutation was mapped to between 63.27 and 67.39 cM. To identify point mutations, genomic DNA between 63.27 and 67.39 cM on chromosome 2 were sequenced. Analysis of sequencing results indicated that the *trin1* mutant was allelic to *abi4*. To verify whether *TRIN1* shared the same genetic locus with *ABI4, trin1* mutant plants were crossed with *abi4-1* which was insensitive to ABA (Finkelstein et al., 1998). All the F_1_ population seeds were insensitive to ABA (Table 1), thus confirming that the *trin1* mutant was indeed allelic to *abi4*. Two changes were found in the coding sequence of *trin1* based on the sequencing results, one was a 3-bp (AAC) deletion at positions 566–568; the other was a single nucleotide change, an A-to-G substitution at position 685 that led to a missense mutation of T to A (Figure 2).

### Analysis of phylogenetic tree of *TRIN1* sequences and phosphorylation sites of trin1 protein

TRIN1/ABI4 is one member of the AP2/ERF family, which can specifically bind to the CE1 element in the promoters of abiotic stress responsive genes and regulate their expression (Mizoi et al., 2012). Homolog of *Arabidopsis TRIN1* has been reported in various plant species, such as *Oryza sativa* and *Zea mays* (Niu et al., 2002). Phylogenetic tree analysis showed that *Arabidopsis TRIN1* was evolutionarily conserved across plant species, whereas no homologs were found in yeast and animals. The closest evolutionary relationship of *TRIN1* was observed between *Capsella rubella* and *Arabidopsis lyrata*. On the other hand, the most distant phylogenetic relationship of *TRIN1* was detected between *Aquilegia coerulea* and *Arabidopsis thaliana* (Supplemental Figure 4).

TOR is a well-known serine/threonine kinase. AZD-insensitivity of *trin1* suggested that TRIN1 likely functioned as a downstream effector of TOR signaling. The phosphorylation of TRIN1 probably relays the TOR signaling cascade in plants. We next asked whether the threonine^230^ (ACT) replaced by an alanine^230^ in trin1 protein affected the phosphorylation status of TRIN1. Online tools were employed to predict the putative phosphorylation sites in TRIN1. Interestingly, threonine^230^ was one of five most possible targets of the upstream kinase (Supplemental Figure 3B). Once threonine^230^ was replaced by alanine^230^, only four putative phosphorylation sites existed in trin1 protein. These results demonstrated that threonine^230^ of TRIN1 likely played a crucial role in TOR signaling transduction in *Arabidopsis*.

### TRIN1 acts as a key player to integrate ABA and TOR signaling during seed-to-seedling transitional stage

**Figure 3:**
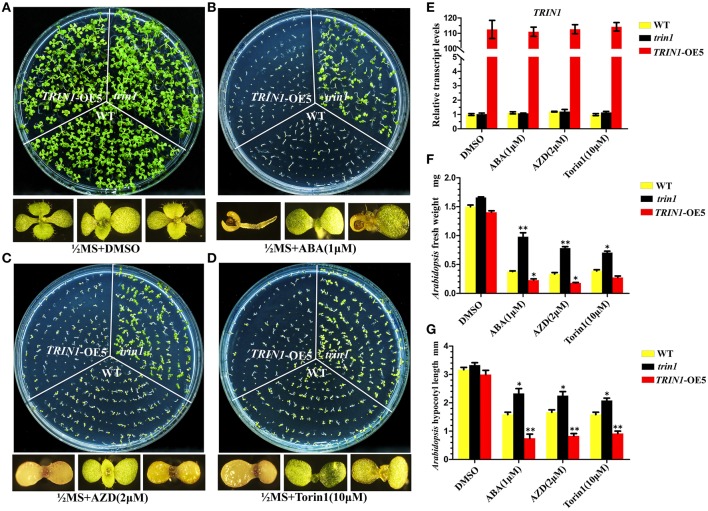
**Seeds of *trin1*, *TRIN1*-OE5, and WT were treated with ABA and mTOR kinase inhibitors**. **(A**–**D)** *trin1, TRIN1*-OE5, and WT seeds were cultured on different medium for 10 days; Enlarged pictures of *TRIN1*-OE5, *trin1*, and WT plants from left to right in the bottom. AZD, ABA, and Torin1 were dissolved in DMSO. **(A)** Using ½ MS containing 1 μg/mL DMSO as controls. **(B–D)** *trin1* plants are insensitive to ABA, AZD and Torin1, whereas *TRIN1*-OE5 plants are sensitive. **(E)** qRT-PCR analysis of *ABI4* transcripts from 10-day-old WT, *trin1*, and *TRIN1*-OE5 plants treated with DMSO, AZD (5 μM), and Torin1 (10 μM) for 36 h. The data represent the mean ±SE of *n* = 3 independent experiments. **(F)** *Arabidopsis* fresh weight of *trin1, TRIN1*-OE5, and WT plants treated as described in **(A–D)**. The data represent the mean ±SE of *n* = 3 independent experiments each containing 30 plants per treatment. Asterisks denote Student's *t-*test significant difference compared with WT plants (^**^P < 0.01). **(G)** *Arabidopsis* shoots length of *trin1, TRIN1*-OE5, and WT plants treated as described in **(A–D)**. The length from aboveground to rosette was measured. The data represents an average of 30 seedlings with three duplicates. Error bars indicate ±SD for triplicates. Asterisks denote Student's *t-*test significant difference compared with WT plants (^*^P < 0.05; ^**^P < 0.01).

To further decipher the functions of TRIN1 in *Arabidopsis*, we generated *TRIN1* overexpression plants by introducing *P35S*::*TRIN1*-HA into WT (Col) *Arabidopsis*. A total of 28 independent transgenic plants were obtained. 21 independent lines showed AZD hypersensitive phenotypes, suggesting that the AZD-sensitive phenotypes resulted from the overexpression of *TRIN1* rather than the T-DNA insertion events. Torin1 has a different structure from that of AZD and is also a well-established TOR inhibitor in *Arabidopsis* (Montané and Menand, 2013; Schepetilnikov et al., 2013), but AZD is a more selective and potent agent against TOR than Torin1. To examine whether *TRIN1* overexpression lines were sensitive to different TOR inhibitors, Torin1 was selected as a parallel control. Five out of twenty-one lines were selected to perform this examination. As expected, these lines showed hyper-sensitive to Torin1 as AZD (Supplemental Figure 5), indicating that different TOR inhibitors can co-target TOR kinase and generate a similar phenotype. *TRIN1* transcriptional levels of these lines were measured. *P35S*::*TRIN1*-OE5 (overexpression line 5) showed the highest *TRIN1* expression level, which was 109-fold higher than that of WT (Figure 3E). Meanwhile, the transcription level of *TRIN1* had no significant difference among DMSO, ABA, AZD, and Torin1 treatments in *P35S*::*TRIN1*-OE5 plants. The lowest *TRIN1* expression level was observed in *P35S*::*TRIN1*-OE1, which was 13-fold higher than that of WT. Importantly, the higher level of *TRIN1* expressed, the more sensitive to AZD showed (Supplemental Figure 5), indicating that the amount of TRIN1 was tightly associated with the sensitivity of AZD in *Arabidopsis*.

Since *TRIN1* is allelic to *ABI4, abi4* was insensitive to ABA (Finkelstein et al., 1998). To determine whether the *trin1* and *P35S*::*TRIN1*-OE5 (*TRIN1*-OE5) seeds were insensitive or sensitive to ABA, the seeds of *trin1, TRIN1*-OE5, and WT were cultured on ½ MS medium supplemented with ABA, and ½ MS medium containing AZD and Torin1 were set as control (Figure 3). On ½ MS + DMSO medium, seedlings growth of *trin1* and *TRIN1*-OE5 was quite similar to WT plants (Figure 3A). However, on ABA medium, *trin1* plants were significantly resistant to ABA but *TRIN1*-OE5 plants were more sensitive to ABA than WT (Figure 3B). In addition, *trin1* seeds could normally germinate and grow on ½ MS medium containing 1 μM ABA, 2 μM AZD, and 10 μM Torin1, whereas *TRIN1*-OE5 plants were overly sensitive to ABA, AZD, and Torin1 (Figures 3B–D). Importantly, the fresh weight of *trin1* seedlings was significantly heavier than that of WT when they were grown on ½ MS medium containing ABA, AZD, or Torin1 (Figure 3F). The shoots length of *trin1* was also significantly increased compared to that of the WT plants on ½ MS medium containing ABA, AZD, or Torin1 (Figure 3G). Together, these results indicated that TRIN1/ABI4 integrated TOR and ABA signaling in regulating plants growth and development in *Arabidopsis*.

### TOR regulates chlorophyll metabolism via TRIN1/ABI4 at the photoautotrophic stage

**Figure 4:**
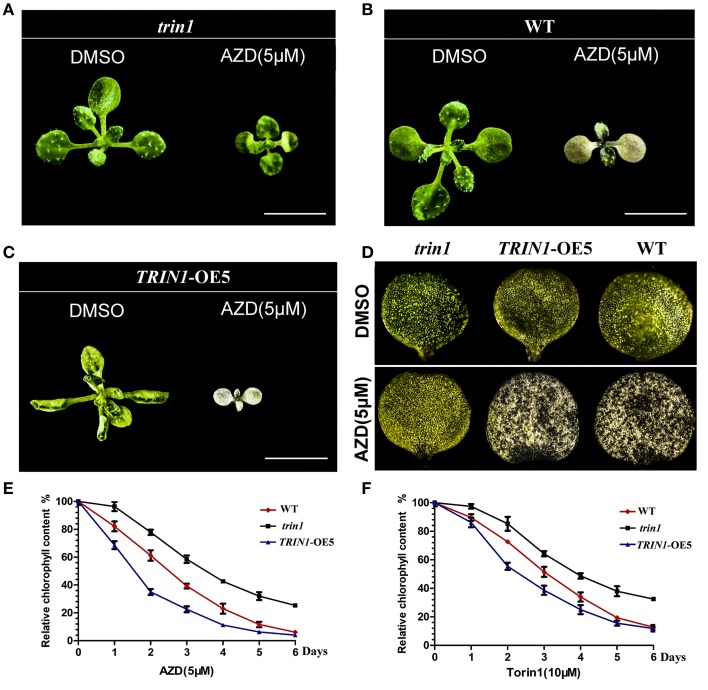
**The effect of AZD on the phenotype of *trin1* mutant and *TRIN1*-OE5 plants**. **(A**–**C)** The phenotype of *trin1, TRIN1*-OE5, and WT plants, 10-day-old seedlings were transferred to ½ MS medium treated for 5 days with or without 5 μM AZD. Bar = 5 mm. **(D)** Images of the first cotyledon of *trin1, TRIN1*-OE5, and WT plants treated as described in **(A–C)** were captured under a stereomicroscope (2.5 × magnification). **(E,F)** Seeds of WT, *trin1*, and *TRIN1*-OE5 were cultured on ½ MS for 7 days. Then, the seedlings were treated for 0–6 days with 5 μM AZD or 10 μM Torin1, and the relative chlorophyll content of plant leaves were measured. The data represents the mean ± SD of *n* = 3 independent experiments.

Cotyledons greening is an extremely important transition from heterotrophism to autrophism in plants. As previously described, inhibition of TOR signaling suppresses the transition from heterotrophism to autrophism in plants (cotyledons cannot turn green). To determine the effect of TOR signaling pathway on chlorophyll metabolism at the photoautotrophic stage, *Arabidopsis* seedlings of *trin1, TRIN1*-OE5, and WT were treated with AZD. AZD interfered with the progress of chlorophyll metabolism, which in turn resulted in that leaves did not retain its green color in WT plants (Figure 4B). *TRIN1*-OE5 plants were more sensitive to AZD than WT plants (Figures 4C,D), whereas *trin1* could rescue the phenotype of AZD inhibition (Figures 4A,D). Relative chlorophyll content of WT, *trin1*, and *TRIN1*-OE5 plant leaves were also measured by using 7-day-old seedlings treated with 5 μM AZD and 10 μM Torin1 for 0–6 days (Figures 4E,F). With time, the chlorophyll contents of WT, *trin1*, and *TRIN1*-OE5 were decreased, whereas *trin1* showed a delayed reduction in chlorophyll content.

**Figure 5:**
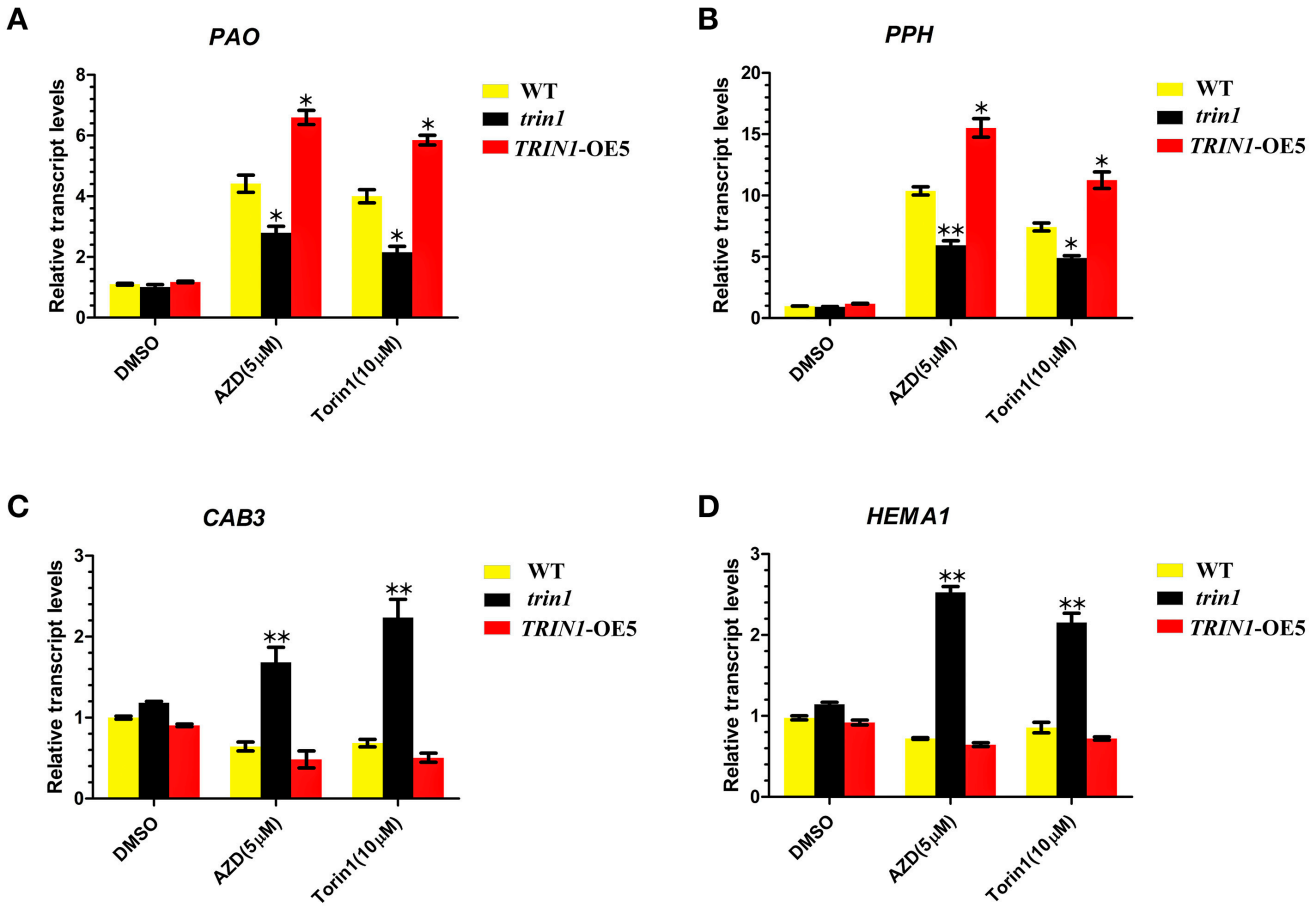
**TOR regulates transcription of genes related to chlorophyll synthesis and decomposition**. **(A–D)** qRT-PCR analysis of *PAO, PPH, CAB3*, and *HEMA1* transcripts in 10- day-old WT, *trin1*, and *TRIN1*-OE5 plants treated with DMSO, AZD (5 μM), and Torin1 (10 μM) for 36 h. The data represents the mean ± SD of *n* = 3 independent experiments. Asterisks denote Student's *t*-test significant difference compared with WT plants (^*^P < 0.05; ^**^P < 0.01).

To further examine the roles of TOR in chlorophyll synthesis and degradation, we analyzed the transcription levels of the key genes associated with chlorophyll synthesis and decomposition using qRT-PCR (Figure 5). The differential expressions of *Pheide a oxygenase* (*PAO*), *Pheophytinase* (*PPH*), *chlorophyll a/b-binding protein 3* (*CAB3*), and *HEMA1* were examined. PAO is a key regulator of chlorophyll catabolism(Pruzinská et al., 2003). PPH is a chloroplast-located hydrolase, which specifically dephytylates the Mg-free chlorophyll pigment and yield pheophorbide (Schelbert et al., 2009). PAO and PPH play important roles in the chlorophyll degradation pathway. The results showed that a significant upregulation of *PAO* mRNA expression in *TRIN1*-OE5 plants compared to that observed in the WT after AZD or Torin1 treatment. On the other hand, *PAO* showed a lower degree of induction after AZD treatment in the *trin1* mutant when compared to WT (Figure 5A). The similar transcript changes of *PPH* were observed in WT, *trin1*, and *TRIN1*-OE5 with AZD or Torin1 treatment (Figure 5B), indicating that TOR and TRIN1 have important roles in the regulation of chlorophyll catabolism. *CAB3*, which is a member of chlorophyll a/b-binding protein family, encodes the most abundant Cab mRNA in developing embryos and young leaves (Leutwiler et al., 1986). *HEMA1* gene encodes glutamyl-tRNA reductase (GluTR) which plays a vital role in chlorophyll biosynthesis in *Arabidopsis thaliana*, (Ilag et al., 1994). Interestingly, the transcript levels of *CAB3* and *HEMA1* were significantly up-regulated with AZD or Torin1 treatment in *trin1* mutant compared to that observed with DMSO (Figures 5C,D). However, transcript levels of *CAB3* and *HEMA1* were down-regulated with AZD or Torin1 treatment in *TRIN1*-OE5 plants compared to that of DMSO (Figures 5C,D). These data indicated that the rate of decomposition of chlorophyll was higher than the synthesis rate, showing that TRIN1 reduced greening of cotyledons and induced chlorophyll degradation by AZD or Torin1 treatment. Therefore, TOR and TRIN1 play important roles in chlorophyll metabolism in *Arabidopsis*.

### TOR signaling regulates the stability of TRIN1/ABI4 protein

**Figure 6:**
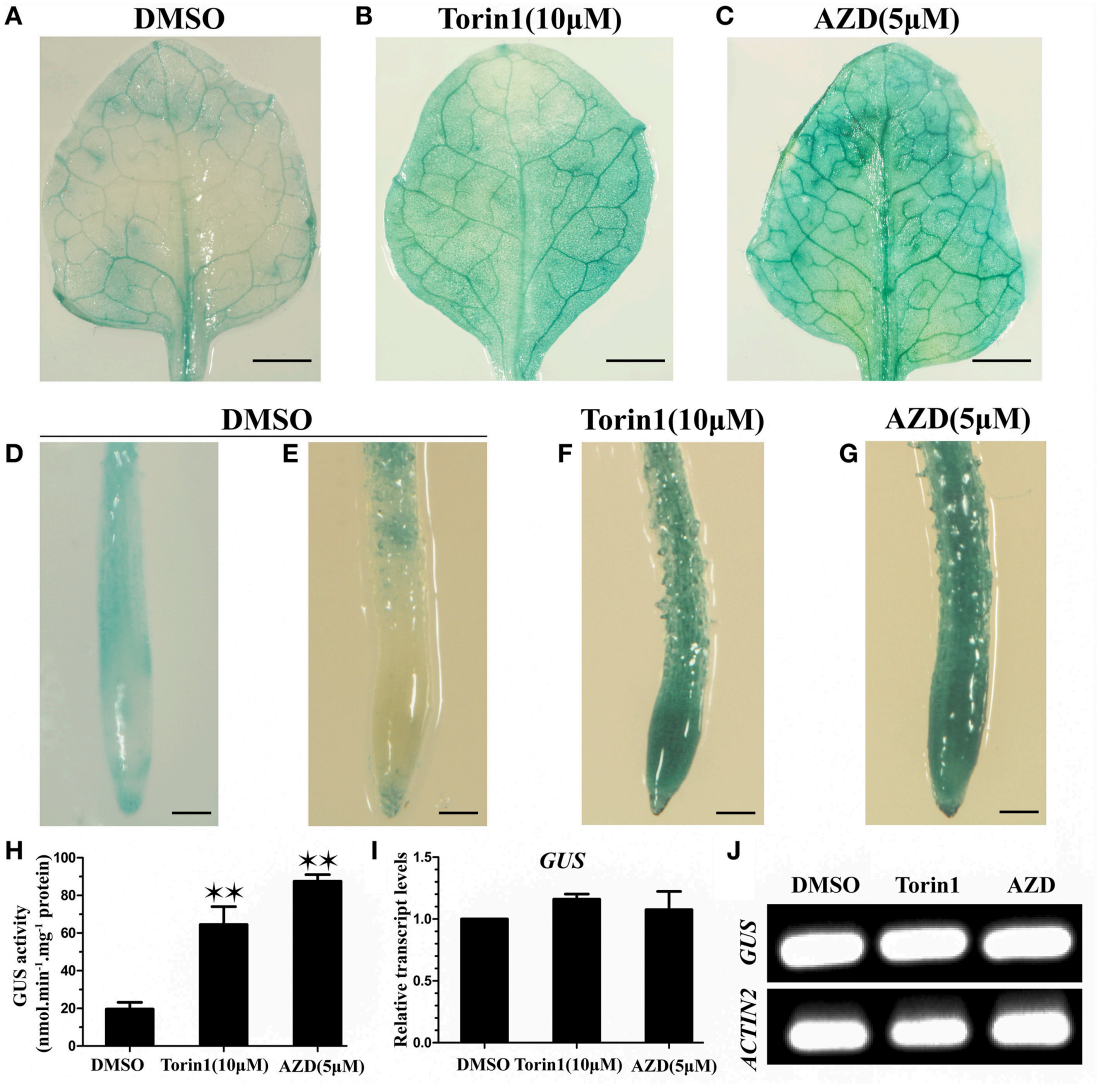
**Suppression of TOR results in the accumulation of TRIN1 protein in *Arabidopsis***. **(A–G)** GUS staining of the first true leaves of *P35S::TRIN1-GUS* OE-2 plants. **(A–C)** GUS staining of leaves of 2-week-old *P35S::TRIN1-GUS* plants treated with DMSO, Torin1, and AZD for 48 h. Bar = 1 mm. **(D)** GUS staining of root tips of 2-week-old *P35S::GUS* plants treated with DMSO for 48 h. **(E–G)** GUS staining of root tips of 2-week-old *P35S::TRIN1-GUS* plants treated with DMSO, Torin1, and AZD for 48 h. Bar = 0.1 mm. **(H)** GUS activity of the first true leaves of 2-week-old *P35S::TRIN1-GUS* plants was measured by fluorometric quantification, plants were treated as described in **(A–C)**. Asterisks denote Student's *t*-test significant difference compared with DMSO (^*^P < 0.05; ^**^P < 0.01). **(I)** qRT-PCR analysis of *GUS* relative transcript level of 2-week-old *PTRIN1::GUS* plants treated with DMSO, AZD (5 μM) and Torin1 (10 μM) for 48 h. The data represent the mean ± SD of *n* = 3 independent experiments. **(J)** RT-PCR analysis of *GUS* transcript level of 2-week-old *PTRIN1::GUS* plants treated as described in **(I)**.

Early studies have shown that the degradation of ABI4 is crucial for its functions in plant growth and development (Gregorio et al., 2014). To determine whether TOR regulates the stability of TRIN1 protein in *Arabidopsis*, we fused *TRIN1* with *GUS* and generated *P35S::TRIN1-GUS* transgenic plants. Seventeen independent transgenic plants were obtained. Nine lines showed AZD hypersensitive phenotype. Three out of nine lines were selected for assessment of GUS activity in leaves (Supplemental Figure 6). The most significant difference in GUS expression level in *P35S::TRIN1-GUS* OE-2 was observed between DMSO and AZD treatments. We respectively examined GUS activity of leaves and root tips of *P35S::TRIN1-GUS* OE-2 plants. High levels of GUS activity were observed in leaves of *P35S::TRIN1-GUS* OE-2 plants treated with TOR inhibitors (Figures 6A–C). Additionally, we quantified the GUS activity in response to DMSO, Torin1, and AZD treatments in the leaves. The quantitative results suggested that the GUS signals were significantly increased when TOR was inhibited by Torin1 or AZD (Figure 6H). Interestingly, GUS signaling was detectable in the root tips but not in the division and elongation regions in *Arabidopsis* root when *P35S::TRIN1-GUS* OE-2 plants growing on ½ MS medium in the absence of TOR inhibitors (Figure 6E), suggesting that that TRIN1 protein can be likely degraded in some plant tissues. Consistently, GUS activity in the roots of *P35S::TRIN1-GUS* OE-2 plants was significantly increased in response to TOR inhibitors treatment (Figures 6D–G). To further examine whether the increasing of GUS signals occurred at transcript level, *GUS* relative transcript levels were analyzed using qRT-PCR and RT-PCR (Figures 6I,J). The *GUS* transcript levels of the *PTRIN1::GUS* plants showed no significant differences among DMSO, Torin1, and AZD treatments, consistent with the previous study (Finkelstein et al., 2011). These results suggested that TOR could likely accelerate the degradation of TRIN1 protein to promote plant growth and development.

## Discussion

### AZD is a highly selective inhibitor of TOR

TOR is a highly conserved Ser/Thr kinase protein. Catalytic domain of *Arabidopsis* TOR shows more than 75% sequence similarity compared with that of *Sc*TOR (yeast TOR) and *Hs*TOR (human TOR; Supplemental Figure 2). Several studies have shown that AZD is able to inhibit TOR kinase activity across species (Calabrese and Blain, 2009; Chresta et al., 2010; Montané and Menand, 2013). The previous studies showed that AZD inhibited TOR kinase activity with a half maximal inhibitory concentration (IC_50_) of 0.8 nM in mammalian cells (Chresta et al., 2010). However, the IC_50_s of AZD for DNA-PK, PI3Kδ and PI3Kα are increased to 1370, 3200, and 3590 nM, respectively (Supplemental Table 3; Chresta et al., 2010). In addition, AZD showed undetectable inhibitory activity against more than 260 different protein kinases even at 10 μM (Chresta et al., 2010). These results demonstrated that AZD acted as a potent and selective mTOR kinase inhibitor with at least 1000-fold specificity over other PI3K or PIKK family members (Chresta et al., 2010). Since TOR belongs to the PI3K superfamily of kinases, it is possible that other PI3Ks are the off-targets of AZD in *Arabidopsis*. However, no homologs of PI3K were found in *Arabidopsis* (Supplemental Table 4), indicating that the off-target effects of AZD were irrelevant in *Arabidopsis*. Recent studies have further shown that AZD displayed potent inhibitory effects on various plants in a dose-dependent manner (Montané and Menand, 2013). Since Torin1 has been widely used as a TOR inhibitor in *Arabidopsis* (Montané and Menand, 2013; Schepetilnikov et al., 2013; Xiong et al., 2013), we therefore examined whether *trin1* or *TRIN1*-OE plants were insensitive or sensitive to Torin1 as the action of AZD. The results showed that both *trin1* and *TRIN1*-OE plants with Torin1 treatment exhibited the same growth pattern as that of AZD (Figure 3). However, Torin1 is the first generation of asTORis with 300-fold selectivity on TOR over other PI3Ks and PIKKs, whereas AZD belongs to the second generation of asTORis with improved pharmacodynamics (Thoreen et al., 2009; Chresta et al., 2010; Caldana et al., 2013; Montané and Menand, 2013; Supplemental Table 3). Our recent observation also showed that the transcriptional profile of AZD-treated *Arabidopsis* was highly overlapping with that of the previous TOR suppression lines generated by independent groups (Ren et al., 2012; Caldana et al., 2013; Xiong et al., 2013; Dong et al., 2015), AZD is therefore the priority selection to minimize the off-targets in this study.

### TRIN1 is a novel effector downstream of TOR signaling

The previous studies showed that ABI4 played an important role in the initiation of plastid retrograde signaling (Acevedo-Hernández et al., 2005; Koussevitzky et al., 2007; León et al., 2012). ABI4 can bind to the promoter of a retrograde-regulated gene *LHCB* through a conserved motif (Koussevitzky et al., 2007). Chloroplast retrograde signaling is required for the initiation and balance Photosynthesis Associated Nuclear Gene (PhANG) expression. ABI4 is a repressor of the PhANG genes such as *LHCB* and *RBCS* in young seedlings and acts as a negative regulator of PhANGs (Acevedo-Hernández et al., 2005; Koussevitzky et al., 2007). Excessive accumulation of the ABI4 protein causes stunted growth in plants. Proteasome regulation of transcriptional regulators has been well-characterized for two ABA response factors: ABI3 and ABI5 (Zhang et al., 2005; Stone et al., 2006). Interestingly, although the ABI4 protein was regulated by post-transcription (Finkelstein et al., 2011; Ludwików, 2015), the degradation of ABI4 protein through ubiquitination remains to be studied. Here, we screened and identified a *trin1* mutant which was insensitive to TOR kinase inhibitors by screening EMS mutagenesis library. Fine mapping and sequencing results showed that the *trin1* mutant was allelic to *abi4*. A point mutation that changed the first base of codon 229 of *trin1* from A to G was identified, resulting in a threonine (ACT) to be replaced by an alanine (GCT; Table 1 and Figure 2). Furthermore, a 3-bp (AAC) deletion at positions 566–568 was found in *trin1*, which resulted in deletion of an asparagine. Interestingly, there were five consecutive asparagines in the TRIN1 protein, but only four consecutive asparagines were existed in *trin1* mutant. It remains to be dissected whether the asparagine deletion results in a change of protein structure or function. In this study we showed that TOR could play an important role in TRIN1/ABI4 degradation. The *trin1* mutant was insensitive to mTOR kinase inhibitors and *TRIN1* overexpression lines were sensitive to mTOR kinase inhibitors (Figures 3C,D), suggesting that TRIN1 maybe function as a regulator or component of the TOR signaling pathway in *Arabidopsis*.

### TOR regulates cotyledons greening via TRIN1/ABI4

**Figure 7:**
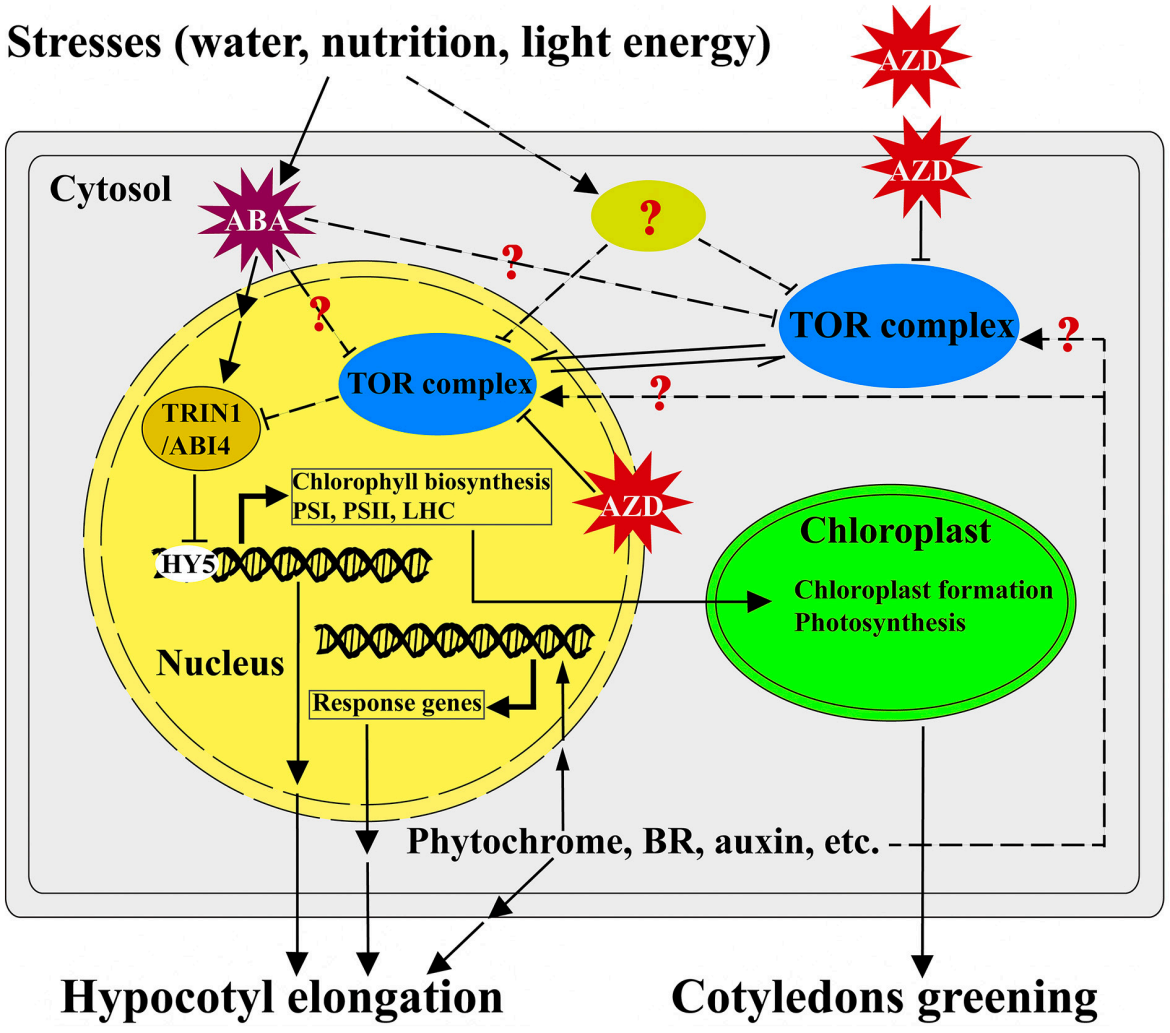
**A working model of TOR-TRIN1 pathway for modulating cotyledons greening**. A simplified model shows how TOR regulates cotyledons greening through TRIN1 in *Arabidopsis*. In this model, AZD is able to suppress the kinase activity of TOR. TOR negatively regulates the stability of the TRIN1/ABI4 protein in nucleus. TRIN1 functions as a negative regulator of chloroplast biogenesis and hypocotyl elongation through inhibiting the activity of Elongated Hypocotyl 5 (HY5). Hypocotyl elongation could be regulated by phytochrome, hormones and other signals might or might not mediate via TOR signaling. Arrows and T-bars represent enhancement and inhibition, respectively. The solid lines indicate the known confirmed or direct interactions. The dashed lines indicate experimentally unproven or indirect links.

Based on the findings of this study, we present a potential working model highlighting the roles of TOR and TRIN1 in regulating the cotyledons greening in *Arabidopsis* (Figure 7). In this model, TOR functions as a master integrator for sensing and signaling the environmental stresses (water, nutrition and light energy stresses; Deprost et al., 2007; Ren et al., 2012; Robaglia et al., 2012; Rexin et al., 2015). Nutrition and energy can activate the activity of TOR while stresses and stress-induced ABA likely suppresses the functions of TOR (Deprost et al., 2007; Ren et al., 2012; Xiong et al., 2013; Rexin et al., 2015; Figure 7). TOR modulates cotyledons greening and hypocotyl elongation in a positive way, whereas TRIN1 acts as a downstream effector of TOR and can mediate TOR signaling to regulate cotyledons greening and hypocotyl elongation in a negative manner (Figure 7).

It should be noted that the early studies showed that interference of TOR could significantly suppress hypocotyl elongation and chlorophyll biosynthesis in *Arabidopsis* (Deprost et al., 2007; Moreau et al., 2012; Ren et al., 2012; Caldana et al., 2013), but little is known about the downstream effectors of TOR to mediate these phenotypes. This is largely due to the early embryo lethality of *tor* mutants which prevents people from screening downstream effectors via classic genetic approaches. In this model, AZD-TOR inducible inhibition system breaks the bottleneck and provides a platform to dissect TOR signaling pathway. For the first time, TRIN1 was identified as the downstream regulator of TOR by using AZD-TOR chemical genetics approach. Interestingly, *TRIN1* is allelic to *ABI4*, which is a key regulator in abscisic acid (ABA) signaling cascade. It is well known that ABA plays pivotal roles in cotyledons greening through activating the expression of ABI4 (Chang and Walling, 1991; Acevedo-Hernández et al., 2005; Yamaguchi-Shinozaki and Shinozaki, 2006). The cotyledons greening regulated by TRIN1 in this study are highly consistent with the functions of ABI4 (Figure 7), indicating TRIN1/ABI4 mediates TOR signaling during cotyledons greening. Hypocotyl elongation is also a complex process which is influenced by phytochrome, Brassinosteriod (BR), auxin, ethylene and other signals (Figure 7). However, little is known about whether these signals modulate hypocotyl elongation via TOR signaling or not (Figure 7). Previous observations show that ABI4 is a negative inhibitor of Elongated Hypocotyl 5 (HY5) which is a key player in regulating the expression of PhANGs and hypocotyl elongation (Susek et al., 1993; McCormac and Terry, 2004; Acevedo-Hernández et al., 2005; Koussevitzky et al., 2007; Jarvis and López-Juez, 2013; Terry and Smith, 2013). Overexpression of ABI4 suppresses HY5 and thus inhibits hypocotyl elongation in *Arabidopsis* (Acevedo-Hernández et al., 2005; Koussevitzky et al., 2007; Finkelstein et al., 2011; Jarvis and López-Juez, 2013; Ludwików, 2015). Altogether, these results demonstrate that TOR regulates cotyledons greening and hypocotyl elongation through TRIN1/ABI4 in *Arabidopsis* (Figure 7). This study provides a platform to dissect functions of TOR signaling cascade in plants. Further dissection of TOR-TRIN1 signaling cascade will significantly advance our understanding of TOR signaling in plants.

## Author contributions

MR, LL, and YS designed the experiments. LL, YS, KW, XZ, PD, ZL, and MR performed the experiments. PD and FL analyzed the data. MR and LL wrote the manuscript.

### Conflict of interest statement

The authors declare that the research was conducted in the absence of any commercial or financial relationships that could be construed as a potential conflict of interest.

## Supplementary material

Supplemental Figure 1 **The vectors of P35S::TRIN1-HA, P35S::TRIN1-GUS, P35S::GUS and PTRIN1::GUS**.

Supplemental Figure 2 **Amino acid sequence alignment of the catalytic domain of TOR in *Saccharomyces cerevisiae, Arabidopsis thaliana* and *Homo sapiens* (http://www.ncbi.nlm.nih.gov/protein/)**. The highly conserved amino acids are highlighted in the red box. The yellow alignment depicts amino acids with 75% similarity.

Supplemental Figure 3 ***At*TOR protein structure and predicted phosphorylation sites of TRIN1 and trin1 protein. (A)** The interaction of *At*TOR domains and RAPTOR, FKBP12, rapamycin (RAP), LST8, and TOR kianse inhibitors (Ku63794, Torin1, and AZD-8055). HEAT repeats: Huntingtin, Elongation factor 3, subunit of protein phosphatase 2A and TOR1; FAT: FRAP, ATM, and TRRAP domain; FRB: FKP12-rapamycin binding domain; FATC: Carboxy-terminal FAT domain; RAPTOR: Regulatory associate protein of TOR; LST8: Lethal with sec-13 protein 8 (Perry and Kleckner, 2003). **(B)** Predicted phosphorylation sites of TRIN1 and trin1 protein. The websites of predicting phosphorylation sites is: http://KinasePhos.mbc.nctu.edu.tw/; a point mutation that results in a change in the first base of codon 229 of *trin1* from A to G was identified, which results in the substitution of threonine (ACT) with an alanine (GCT), we predicted the threonine sites of TRIN1 and trin1 protein. The different sites of TRIN1 and trin1 were marked with a red oval and rectangle, respectively. Predicted phosphorylation sites of the TRIN1 protein is presented at the left, and the right panel shows phosphorylation sites of the trin1 protein.

Supplemental Figure 4 **Phylogenetic tree of the *TRIN1* sequences of 30 taxa**. *TRIN1* sequences were collected from different source databases (Phytozome http://www.phytozome.net, version 8.0; Plaza http://bioinformatics.psb.ugent.be/plaza/ version 2.5 and NCBI Genbank http://www.ncbi.nlm.nih.gov/genbank/) based on sequence similarity with *Arabidopsis thaliana TRIN1* gene (Wind et al., 2013). The evolutionary history was inferred using the UPGMA method. The tree is drawn to scale, with branch lengths in the same units as those of the evolutionary distances used to infer the phylogenetic tree. The evolutionary distances were computed using the maximum composite likelihood method and are in the units of the number of base substitutions per site. The analysis involved 33 nucleotide sequences. Evolutionary analyses were conducted in MEGA5.

Supplemental Figure 5 ***P35S*::*TRIN1* overexpression lines were sensitive to AZD. (A**–**C)** *P35S*::*TRIN1* overexpression lines and WT seeds were cultured on ½ medium containing DMSO, 1 μM AZD and 10 μM Torin1 for 15 days. Images of representative seedlings were captured. **(D)** RT-PCR analysis of *TRIN1* transcript level of 2-week-old *P35S*::*TRIN1* overexpression lines. The name of lanes is the same as **(A)** from left to right.

Supplemental Figure 6 **The GUS activity of *P35S*::*TRIN1*-*GUS* overexpression lines**. GUS staining of the first true leaves of 2-week-old *P35S*::*TRIN1*-*GUS* lines treated with DMSO and 5 μM AZD for 48 h.

Supplemental Table 1 **Primers for cloning promoter or full-length CDS**.

Supplemental Table 2 **Primers for quantitative real-time PCR**.

Supplemental Table 3 **The targets of AZD8055 and Torin1 and their IC50**.

Supplemental Table 4 **The comparison of the homologs of PI3Ks between animals and plants**.
