# Supplementary figures and images for: TOR-inhibitor insensitive-1 (TRIN1) regulates cotyledons greening in Arabidopsis
Source: Front Plant Sci. 2015 Oct 19;6:861. doi: 10.3389/fpls.2015.00861 (PMC4617058; doi:10.3389/fpls.2015.00861)

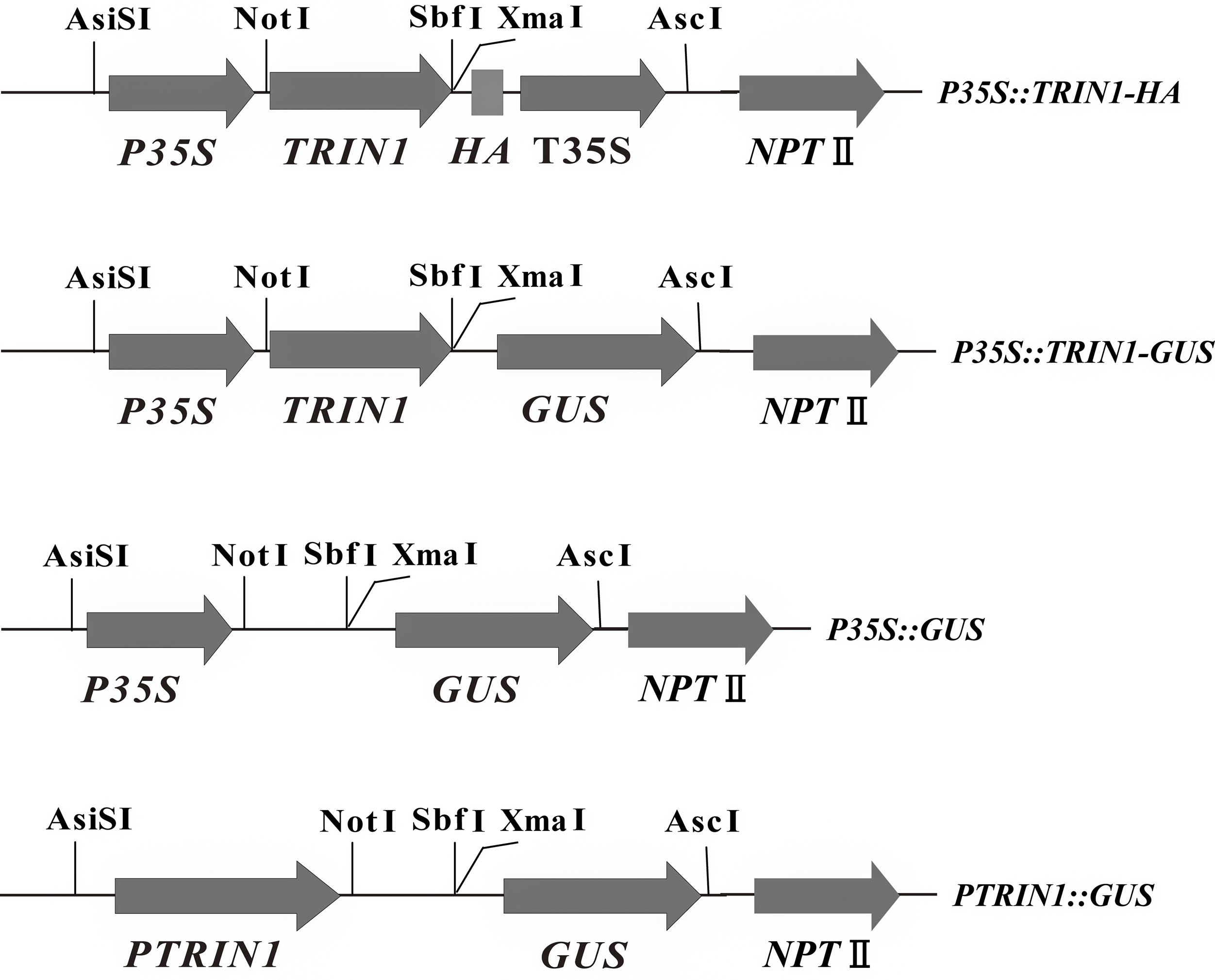

Supplement: Supplemental Figure 1 — The vectors of P35S::TRIN1-HA, P35S::TRIN1-GUS, P35S::GUS and PTRIN1::GUS. [file Image1.TIF]

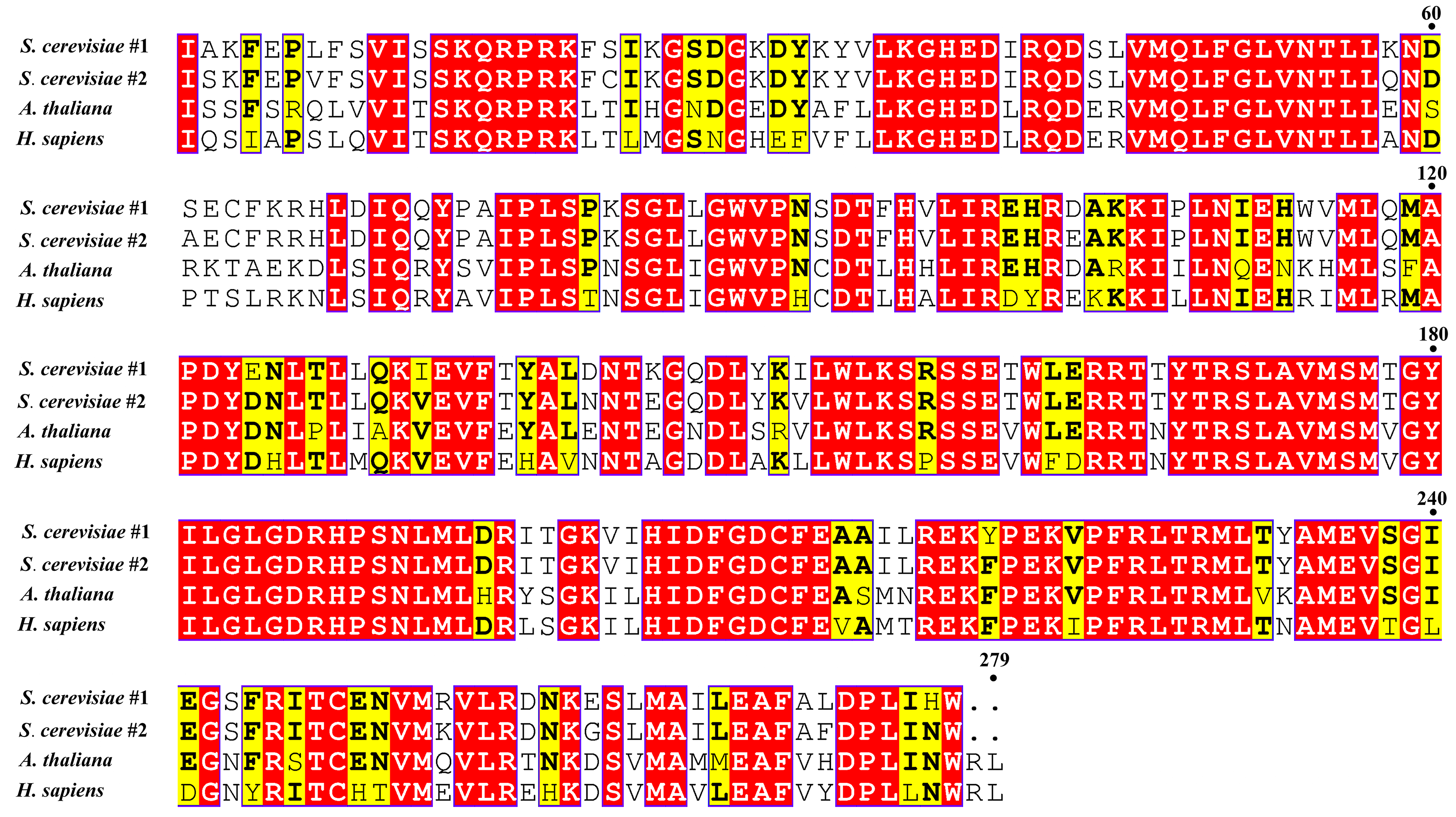

Supplement: Supplemental Figure 2 — Amino acid sequence alignment of the catalytic domain of TOR in Saccharomyces cerevisiae, Arabidopsis thaliana and Homo sapiens (http://www.ncbi.nlm.nih.gov/protein/). The highly conserved amino acids are highlighted in the red box. The yellow alignment depicts amino acids with 75% similarity. [file Image2.TIF]

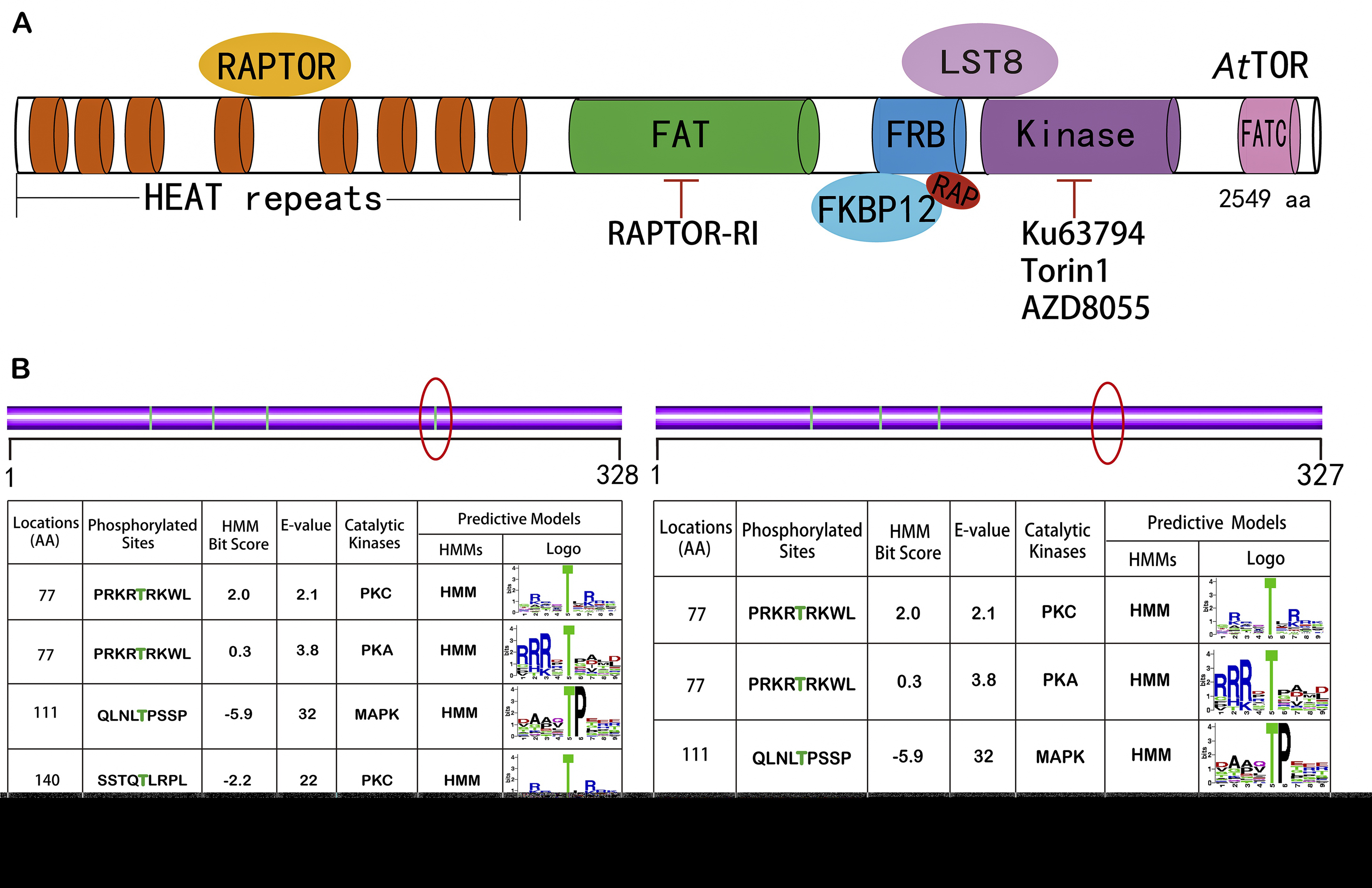

Supplement: Supplemental Figure 3 — AtTOR protein structure and predicted phosphorylation sites of TRIN1 and trin1 protein. (A) The interaction of AtTOR domains and RAPTOR, FKBP12, rapamycin (RAP), LST8, and TOR kianse inhibitors (Ku63794, Torin1, and AZD-8055). HEAT repeats: Huntingtin, Elongation factor 3, subunit of protein phosphatase 2A and TOR1; FAT: FRAP, ATM, and TRRAP domain; FRB: FKP12-rapamycin binding domain; FATC: Carboxy-terminal FAT domain; RAPTOR: Regulatory associate protein of TOR; LST8: Lethal with sec-13 protein 8 (Perry and Kleckner, 2003). (B) Predicted phosphorylation sites of TRIN1 and trin1 protein. The websites of predicting phosphorylation sites is: http://KinasePhos.mbc.nctu.edu.tw/; a point mutation that results in a change in the first base of codon 229 of trin1 from A to G was identified, which results in the substitution of threonine (ACT) with an alanine (GCT), we predicted the threonine sites of TRIN1 and trin1 protein. The different sites of TRIN1 and trin1 were marked with a red oval and rectangle, respectively. Predicted phosphorylation sites of the TRIN1 protein is presented at the left, and the right panel shows phosphorylation sites of the trin1 protein. [file Image3.TIF]

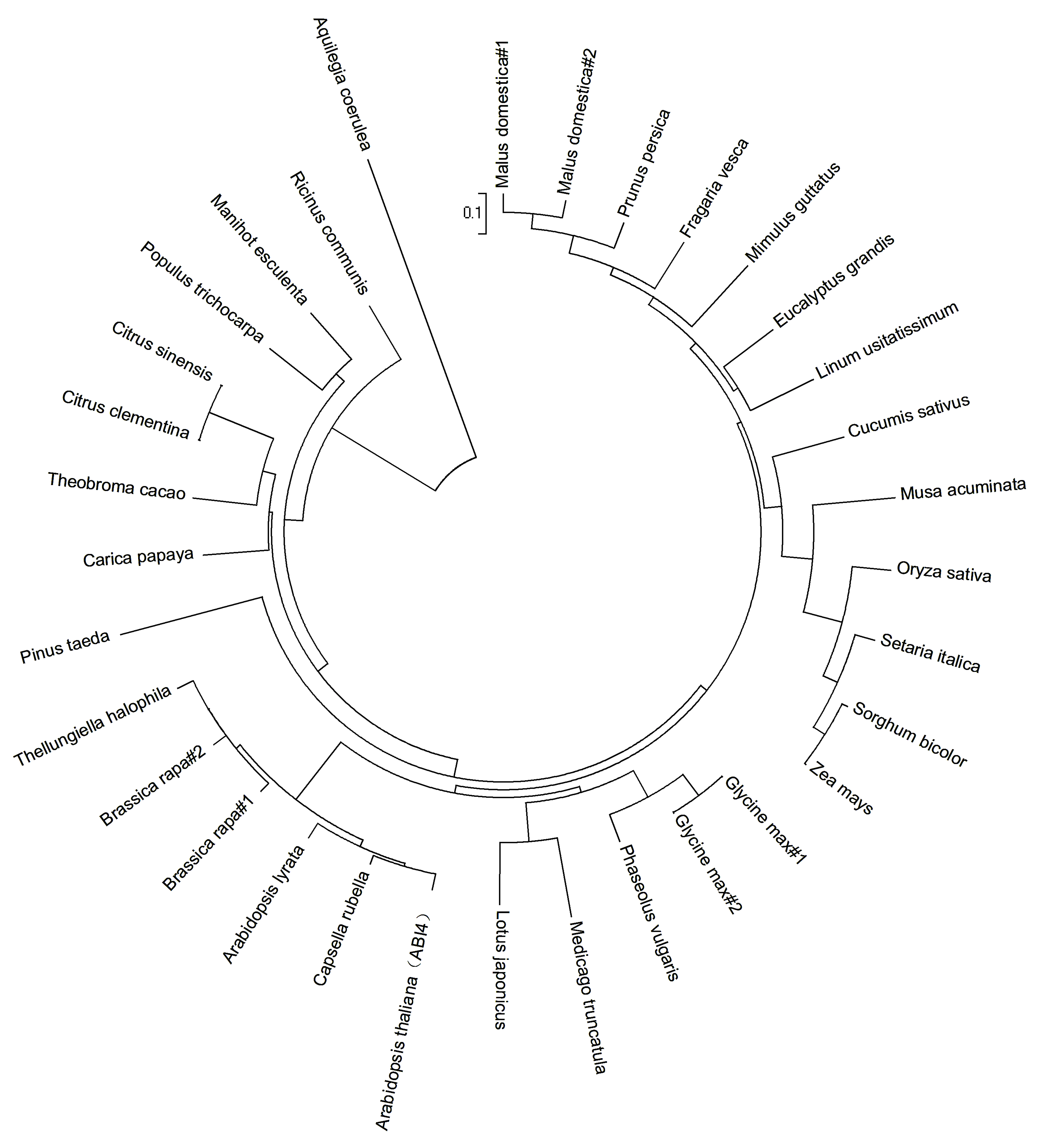

Supplement: Supplemental Figure 4 — Phylogenetic tree of the TRIN1 sequences of 30 taxa. TRIN1 sequences were collected from different source databases (Phytozome http://www.phytozome.net, version 8.0; Plaza http://bioinformatics.psb.ugent.be/plaza/ version 2.5 and NCBI Genbank http://www.ncbi.nlm.nih.gov/genbank/) based on sequence similarity with Arabidopsis thaliana TRIN1 gene (Wind et al., 2013). The evolutionary history was inferred using the UPGMA method. The tree is drawn to scale, with branch lengths in the same units as those of the evolutionary distances used to infer the phylogenetic tree. The evolutionary distances were computed using the maximum composite likelihood method and are in the units of the number of base substitutions per site. The analysis involved 33 nucleotide sequences. Evolutionary analyses were conducted in MEGA5. [file Image4.TIF]

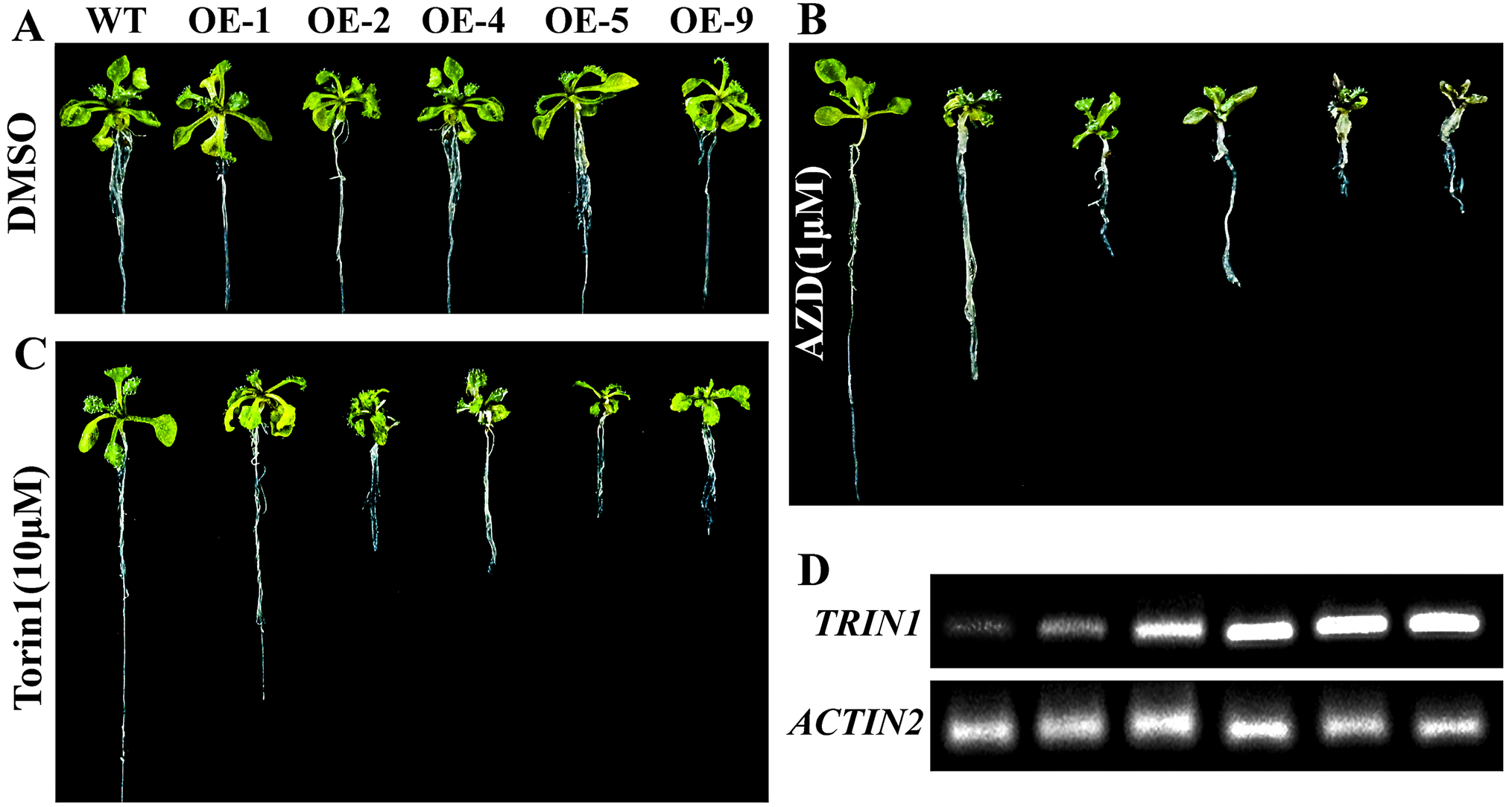

Supplement: Supplemental Figure 5 — P35S::TRIN1 overexpression lines were sensitive to AZD. (A–C) P35S::TRIN1 overexpression lines and WT seeds were cultured on ½ medium containing DMSO, 1 μM AZD and 10 μM Torin1 for 15 days. Images of representative seedlings were captured. (D) RT-PCR analysis of TRIN1 transcript level of 2-week-old P35S::TRIN1 overexpression lines. The name of lanes is the same as (A) from left to right. [file Image5.TIF]

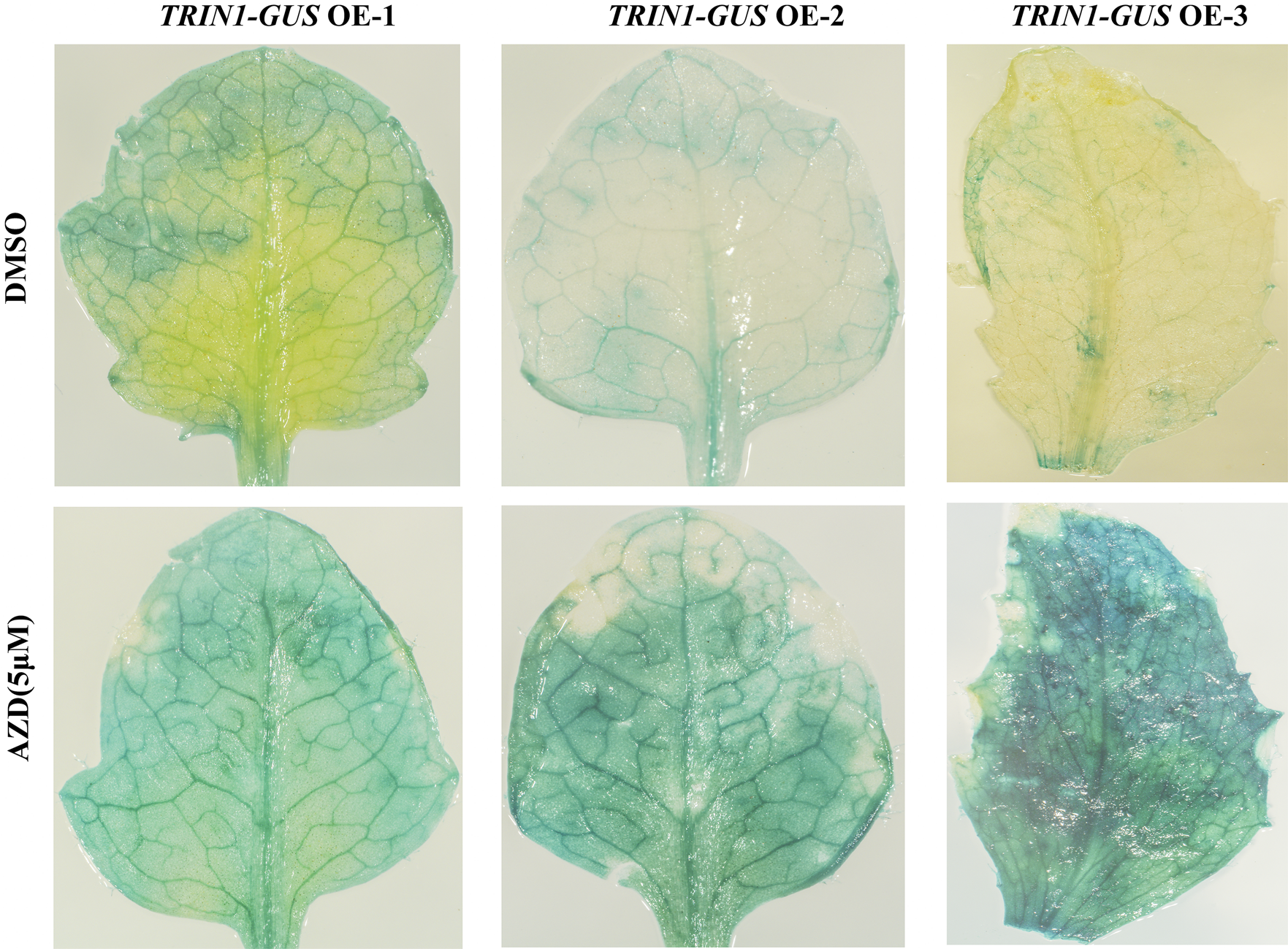

Supplement: Supplemental Figure 6 — The GUS activity of P35S::TRIN1-GUS overexpression lines. GUS staining of the first true leaves of 2-week-old P35S::TRIN1-GUS lines treated with DMSO and 5 μM AZD for 48 h. [file Image6.TIF]
