# Supplementary material for: TOR-inhibitor insensitive-1 (TRIN1) regulates cotyledons greening in Arabidopsis
Source: Front Plant Sci. 2015 Oct 19;6:861. doi: 10.3389/fpls.2015.00861 (PMC4617058; doi:10.3389/fpls.2015.00861)

***Supplementary Material***

**TOR-inhibitor insensitive-1 (TRIN1)** **regulates cotyledons greening in** ***Arabidopsis***

Linxuan Li^+,1^, Yun Song^+,2^, Kai Wang^1^, Pan Dong^1^, Xueyan Zhang^2^, Fuguang Li^2^, Zhengguo Li^1^, Maozhi Ren^*,1^

^1^School of Life Sciences, Chongqing University, Chongqing, China

^2^Institute of Cotton Research, Chinese Academy of Agricultural Sciences, the State Key Laboratory of Cotton Biology, Henan, China

^+^Both authors contributed equally to this work

^*^Correspondence:

Maozhi Ren

School of Life Sciences

Chongqing University

174 Shazheng ST, Shapingba,

Chongqing, China, 400045

Phone: 86-13527313471

E-mail: [renmaozhi@cqu.edu.cn](mailto:renmaozhi@cqu.edu.cn)

**Supplemental Table 1 ǀ Primers for cloning promoter or full-length CDS.**

| Primers | Sequences | Length |
| --- | --- | --- |
| *PTRIN1* F  *PTRIN1* R  *TRIN1* F  *TRIN1* R | 5’- GCGATCGCACGGTAGATTAGTCTTTCTAGGCG-3’  5’- GCGGCCGCGCGAAGAGGAAGAGGAAGTAGAG-3’  5’- GCGGCCGCATGGACCCTTTAGCTTCCCAAC-3’  5’- CCTGCAGGATAGAATTCCCCCAAGATGGG-3’ | 32  31  30  29 |
| *GUS* F | 5’- CCCGGGATGTTACGTCCTGTAGAAACCCC-3’ | 29 |
| *GUS* R | 5’- GGCGCGCCTCATTGTTTGCCTCCCTGCTGCGG-3’ | 32 |


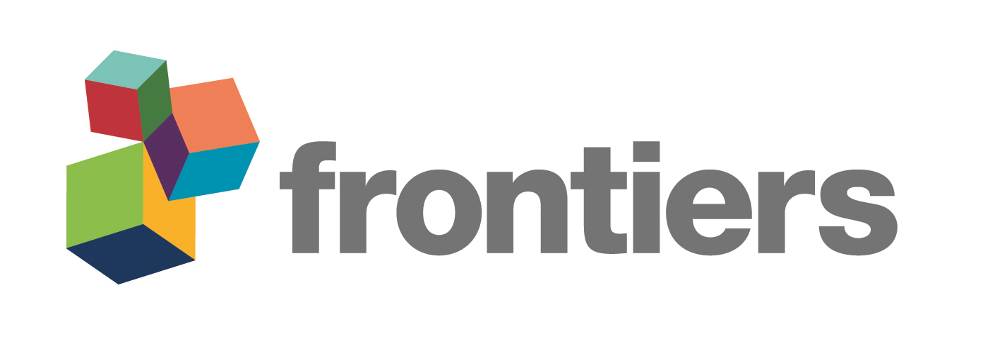

Supplement: Supplemental Table 1 — Primers for cloning promoter or full-length CDS. [file Table1.DOCX]
