# Supplementary material for: TOR-inhibitor insensitive-1 (TRIN1) regulates cotyledons greening in Arabidopsis
Source: Front Plant Sci. 2015 Oct 19;6:861. doi: 10.3389/fpls.2015.00861 (PMC4617058; doi:10.3389/fpls.2015.00861)

***Supplementary Material***

**TOR-inhibitor insensitive-1 (TRIN1)** **regulates cotyledons greening in** ***Arabidopsis***

Linxuan Li^+,1^, Yun Song^+,2^, Kai Wang^1^, Pan Dong^1^, Xueyan Zhang^2^, Fuguang Li^2^, Zhengguo Li^1^, Maozhi Ren^*,1^

^1^School of Life Sciences, Chongqing University, Chongqing, China

^2^Institute of Cotton Research, Chinese Academy of Agricultural Sciences, the State Key Laboratory of Cotton Biology, Henan, China

+Both authors contributed equally to this work

^*^Correspondence:

Maozhi Ren

School of Life Sciences

Chongqing University

174 Shazheng ST, Shapingba,

Chongqing, China, 400045

Phone: 86-13527313471

E-mail: [renmaozhi@cqu.edu.cn](mailto:renmaozhi@cqu.edu.cn)

**Supplemental Table 2 ǀ Primers for quantitative real-time PCR.**

| Primers | Sequences | Length |
| --- | --- | --- |
| *TRIN1* F | 5’-GCGTTAGGGCAGGAACAAGG-3’ | 20 |
| *TRIN1* R | 5’-TCCAGACCCATAGAACATACCG-3’ | 20 |
| *PAO* F | 5’ -TGCGAATGATGACAGTCCAA-3’ | 20 |
| *PAO* R | 5’-TGAGCAAATCCAAATGACCC-3’ | 20 |
| *PPH* F | 5’-CTCACTGCGAGTGGAAGCC-3’ | 19 |
| *PPH* R  *HEMA1* F  *HEMA1* R  *CAB3* F  *CAB3* R  *GUS* F  *GUS* R  *ACTIN2* F  *ACTIN2* R | 5’-GAAATGAACCAACGCCAAA-3’  5’-AAGTGCTTTCGTTGGTTGTC-3’  5’-GACCCAATGGTGTCTGTGAA-3’  5’-GGACTTGCTTTACCCCGGTG-3’  5’-TCGGTAGCAAGACCCAATGG-3’  5’-AGACTGTAACCACGCGTCTG-3’  5’-ACTTGCAAAGTCCCGCTAGT-3’  5’-GCACTTGCACCAAGCAGCAT-3’  5’-CCTTTCAGGTGGTGCAACGAC-3’ | 19  20  20  20  20  20  20  20  21 |


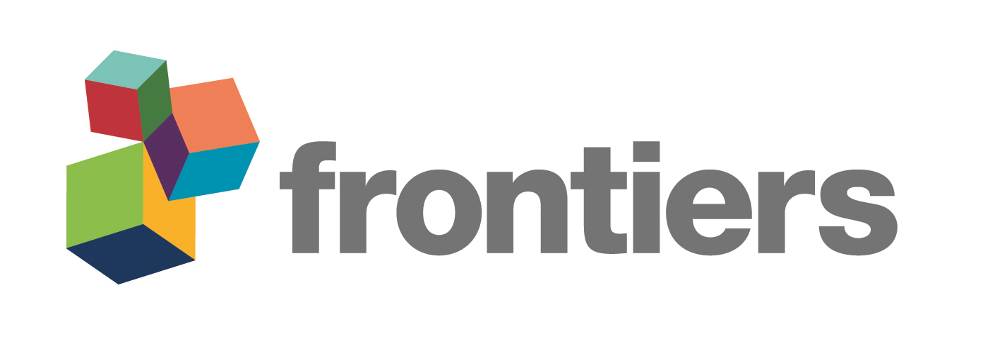

Supplement: Supplemental Table 2 — Primers for quantitative real-time PCR. [file Table2.DOCX]
