# Supplementary material for: TOR-inhibitor insensitive-1 (TRIN1) regulates cotyledons greening in Arabidopsis
Source: Front Plant Sci. 2015 Oct 19;6:861. doi: 10.3389/fpls.2015.00861 (PMC4617058; doi:10.3389/fpls.2015.00861)

***Supplementary Material***

**TOR-inhibitor insensitive-1 (TRIN1)** **regulates cotyledons greening in** ***Arabidopsis***

Linxuan Li^+,1^, Yun Song^+,2^, Kai Wang^1^, Pan Dong^1^, Xueyan Zhang^2^, Fuguang Li^2^, Zhengguo Li^1^, Maozhi Ren^*,1^

^1^School of Life Sciences, Chongqing University, Chongqing, China

^2^Institute of Cotton Research, Chinese Academy of Agricultural Sciences, the State Key Laboratory of Cotton Biology, Henan, China

+Both authors contributed equally to this work

^*^Correspondence:

Maozhi Ren

School of Life Sciences

Chongqing University

174 Shazheng ST, Shapingba,

Chongqing, China, 400045

Phone: 86-13527313471

E-mail: [renmaozhi@cqu.edu.cn](mailto:renmaozhi@cqu.edu.cn)

**Supplemental Table 3 ǀ The targets of AZD8055 and Torin1 and their IC50.**

| Target | ValueName | AZD8055 | Torin1 |
| --- | --- | --- | --- |
|  |  | Value | Value |
| mTOR (truncated) | IC50 | 0.13 nM | — |
| mTOR (full length) | IC50 | 0.8 nM | 4.32 nM |
| DNA-PK | IC50 | 1,370 nM | 6.34 nM |
| PI3Kδ/p110δ | IC50 | 3,200 nM] | 564 nM |
| PI3Kα/p110α | IC50 | 3,590 nM | 250 nM |
| ATM | IC50 | 7,350 nM | — |
| PI3Kβ | IC50 | 18,900 nM | — |
| PI3Kγ/p110γ | IC50 | >14,790 nM | 171 nM |
| mTORC1 | IC50 | — | 2 nM |
| mTORC2 | IC50 | — | 10 nM |
| C2α | IC50 | — | 176 nM |
| hVPS34 | IC50 | — | 533 nM |
| C2β | IC50 | — | 549 nM |
| PI4Kβ | IC50 | — | 6,680 nM |
| PI4Kα | IC50 | — | >10 μM |

These data come from Selleck.cn (<http://www.selleck.cn/>). The targets of AZD8055 and Torin1 and the values of IC50 come from previous studies results (Thoreen et al., 2009; Chresta et al., 2010; Liu et al., 2010).

**Supplementary references**

Chresta, C.M., Davies, B.R., Hickson, I., Harding, T., Cosulich, S., Critchlow, S.E., Vincent, J.P., Ellston, R., Jones, D., Sini, P., James, D., Howard, Z., Dudley, P., Hughes, G., Smith, L., Maguire, S., Hummersone, M., Malagu, K., Menear, K., Jenkins, R., Jacobsen, M., Smith, G.C., Guichard, S., and Pass, M. (2010). AZD8055 is a potent, selective, and orally bioavailable ATP-competitive mammalian target of rapamycin kinase inhibitor with in vitro and in vivo antitumor activity. *Cancer Res* 70**,** 288-298.

Liu, Q., Chang, J.W., Wang, J., Kang, S.A., Thoreen, C.C., Markhard, A., Hur, W., Zhang, J., Sim, T., Sabatini, D.M., and Gray, N.S. (2010). Discovery of 1-(4-(4-propionylpiperazin-1-yl)-3 -(trifluoromethyl)phenyl)-9-(quinolin-3-yl)benz o[h][1,6]naphthyridin-2(1H)-one as a highly potent, selective mammalian target of rapamycin (mTOR) inhibitor for the treatment of cancer. *J Med Chem* 53**,** 7146-7155.

Thoreen, C.C., Kang, S.A., Chang, J.W., Liu, Q.S., Zhang, J.M., Gao, Y., Reichling, L.J., Sim, T.B., Sabatini, D.M., and Gray, N.S. (2009). An ATP-competitive Mammalian Target of Rapamycin Inhibitor Reveals Rapamycin-resistant Functions of mTORC1. *Journal of Biological Chemistry* 284**,** 8023-8032.


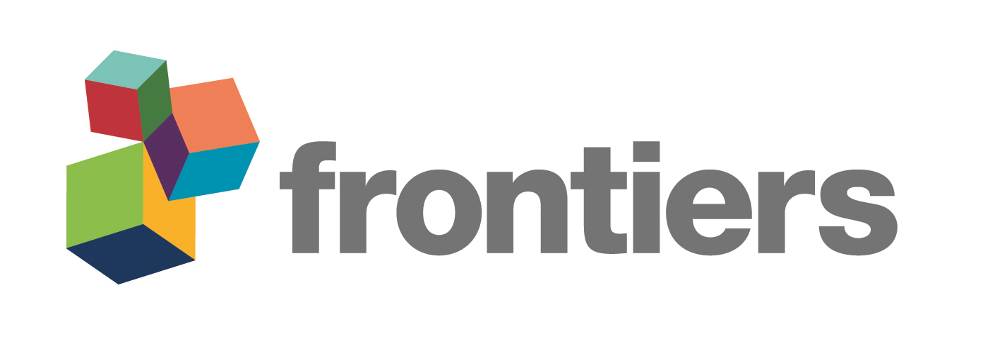

Supplement: Supplemental Table 3 — The targets of AZD8055 and Torin1 and their IC50. [file Table3.DOC]
