# Supplementary material for: TOR-inhibitor insensitive-1 (TRIN1) regulates cotyledons greening in Arabidopsis
Source: Front Plant Sci. 2015 Oct 19;6:861. doi: 10.3389/fpls.2015.00861 (PMC4617058; doi:10.3389/fpls.2015.00861)

***Supplementary Material***

**TOR-inhibitor insensitive-1 (TRIN1)** **regulates cotyledons greening in** ***Arabidopsis***

Linxuan Li^+,1^, Yun Song^+,2^, Kai Wang^1^, Pan Dong^1^, Xueyan Zhang^2^, Fuguang Li^2^, Zhengguo Li^1^, Maozhi Ren^*,1^

^1^School of Life Sciences, Chongqing University, Chongqing, China

^2^Institute of Cotton Research, Chinese Academy of Agricultural Sciences, the State Key Laboratory of Cotton Biology, Henan, China

+Both authors contributed equally to this work

^*^Correspondence:

Maozhi Ren

School of Life Sciences

Chongqing University

174 Shazheng ST, Shapingba,

Chongqing, China, 400045

Phone: 86-13527313471

E-mail: [renmaozhi@cqu.edu.cn](mailto:renmaozhi@cqu.edu.cn)

**Supplemental Table 4 ǀ The comparison of the homologs of PI3Ks between animals**

**and plants.**

| Subunits  Species | DNA-PK | p110γ | C2α | PI3Kδ | PI3Kα |
| --- | --- | --- | --- | --- | --- |
| *Mus musculus* | 1 | 3 | 1 | 5 | 1 |
| *Homo sapiens* | 1 | 4 | 1 | 1 | 1 |
| *Arabidopsis thaliana* | - | - | - | - | - |
| *Oryza sativa* | - | - | - | - | - |
| *Zea mays* | - | - | - | - | - |
| *Brassica campestris* | - | - | - | - | - |


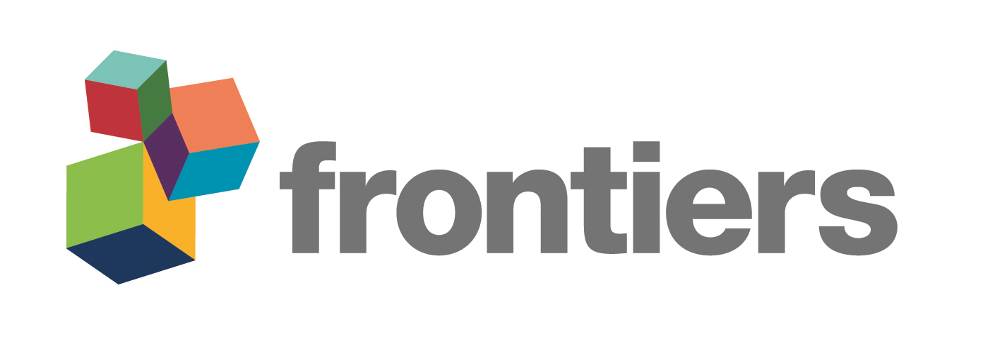

Supplement: Supplemental Table 4 — The comparison of the homologs of PI3Ks between animals and plants. [file Table4.DOCX]
